# The circadian E3 ligase complex SCF^FBXL3+CRY^ targets TLK2

**DOI:** 10.1038/s41598-018-36618-3

**Published:** 2019-01-17

**Authors:** Stephanie Papp Correia, Alanna B. Chan, Megan Vaughan, Norjin Zolboot, Valerie Perea, Anne-Laure Huber, Anna Kriebs, James J. Moresco, John R. Yates, Katja A. Lamia

**Affiliations:** Department of Molecular Medicine, The Scripps Research Institute, 10550 North Torrey Pines Road, La Jolla, CA 92037 USA

## Abstract

We recently demonstrated that the circadian clock component CRY2 is an essential cofactor in the SCF^FBXL3^-mediated ubiquitination of c-MYC. Because our demonstration that CRY2 recruits phosphorylated substrates to SCF^FBXL3^ was unexpected, we investigated the scope of this role by searching for additional substrates of FBXL3 that require CRY1 or CRY2 as cofactors. Here, we describe an affinity purification mass spectrometry (APMS) screen through which we identified more than one hundred potential substrates of SCF^FBXL3+CRY1/2^, including the cell cycle regulated Tousled-like kinase, TLK2. Both CRY1 and CRY2 recruit TLK2 to SCF^FBXL3^, and TLK2 kinase activity is required for this interaction. Overexpression or genetic deletion of CRY1 and/or CRY2 decreases or enhances TLK2 protein abundance, respectively. These findings reinforce the idea that CRYs function as co-factors for SCF^FBXL3^, provide a resource of potential substrates, and establish a molecular connection between the circadian and cell cycle oscillators via CRY-modulated turnover of TLK2.

## Introduction

Circadian clocks are present in virtually all cells in the body and maintain an intrinsic 24-hour period that allows organisms to predict recurring daily changes in their environment^1^. The core of the mammalian clock is composed of a transcriptional and translational negative feedback loop, in which the transcription factors CLOCK and BMAL1 heterodimerize and promote the expression of their own repressors cryptochrome (*Cry1&2*) and period (*Per1-3)*^2,3^. The clock is reset by several post-translational modifications, including ubiquitination and subsequent degradation of CRY1 and CRY2 in the nucleus by the SKP-CULLIN-FBOX (SCF) family E3 ligase containing the F-box and leucine rich repeat (FBXL3) substrate adaptor (SCF^FBXL3^)^4–6^. AMP-activated protein kinase (AMPK)-mediated phosphorylation of CRY1 promotes its association with SCF^FBXL3^, and subsequent degradation^7^.

In addition to the function of CRYs as transcriptional repressors in the circadian clock, we recently discovered a previously unappreciated role for CRY2 as an essential cofactor in the SCF^FBXL3^ mediated ubiquitination of the oncoprotein c-MYC, which may contribute to the mechanistic basis underlying enhanced tumorigenesis in the context of disrupted circadian rhythms^8^. Here, we describe an approach using affinity purification mass spectrometry (APMS) to identify additional substrates recruited to SCF^FBXL3^ by CRY1 or CRY2. We found that SCF^FBXL3+CRY1/2^ interacts with and destabilizes the Tousled-like kinase, TLK2.

TLK1 and TLK2 are highly homologous cell cycle regulated kinases with peak activity in S phase^9^. They were initially characterized after their plant homolog Tousled (TSL) was found to be necessary for proper flower and leaf formation, likely by regulating cell division during development^9–12^. TLK1 and TLK2 regulate chromatin assembly by phosphorylating ASF1 histone chaperone proteins^13–15^ and are catalytically activated and targeted for proteasomal degradation in response to DNA damage^9,13^. TLK2 is uniquely important for checkpoint recovery in response to DNA damage^15^. Additionally, TLK2 deregulation has been linked to cancer in multiple studies^16–18^. Our finding that SCF^FBXL3+CRY1/2^ enhances turnover of TLK2 suggests another mechanistic link by which deregulation of clock components could lead to a cancer-prone phenotype.

## Results

### Search for targets of SCF^FBXL3+CRY1/2^

**Figure 1:**
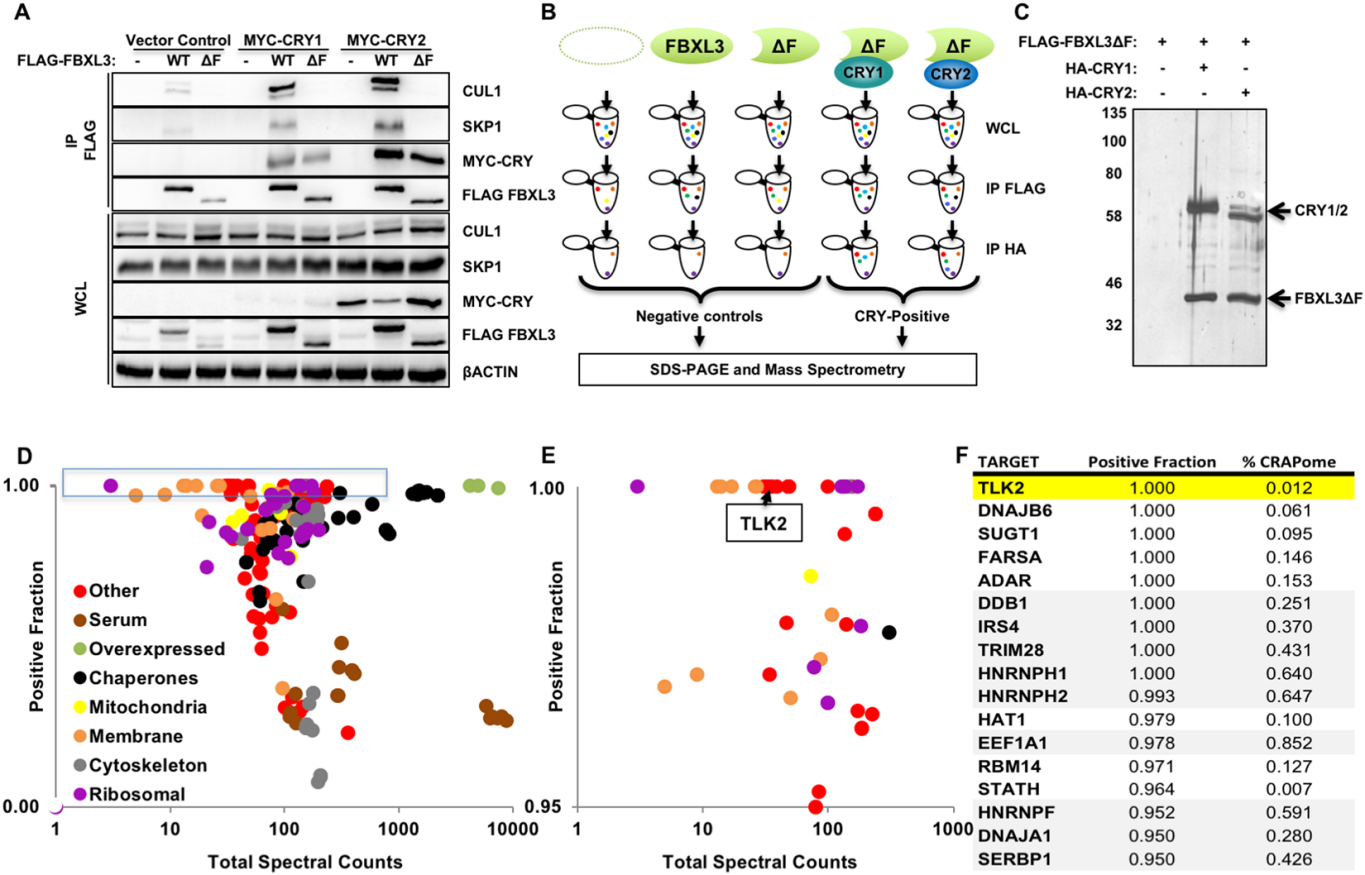
Unbiased screen for FBXL3 partners recruited by CRY1 and/or CRY2. (**A**) CUL1, SKP1, βACTIN, MYC-CRY1, MYC-CRY2, FLAG-FBXL3 detected by immunoblot (IB) in whole cell lysate (WCL) or following immunoprecipitation (IP) of FLAG tagged proteins from cells transiently expressing the indicated plasmids. (**B**) Screening conditions and workflow. (**C**) Proteins detected by silver stain in samples prepared as shown in (**B**). Molecular weight markers (kDa) at left. Uncropped gel in Supplementary Fig. 4. (**D**) Each symbol represents one of 141 proteins for which at least 30 peptides were detected in CRY-positive samples. Colors denote association with subcellular compartments or protein complexes as indicated. (**E**) Magnified view of the data in the blue rectangle in (**D**). (**F**) List of top candidates from the screen, with TLK2 highlighted in yellow. Gray shading indicates proteins frequently detected my mass spectrometry in Flag IPs from 293 T cells. In (**D,E**) Total spectral counts indicates the number of peptides detected in all samples in all three experiments combined. Positive fraction indicates the number of peptides detected in CRY-positive samples divided by the number detected in all samples.

Following our identification of a CRY and SCF^FBXL3^ complex that recruits c-MYC for ubiquitination, we performed a proteomic screen to identify additional substrates, using truncated FBXL3 lacking the F-box (FBXL3ΔF) to prevent its association with the SKP-CULLIN E3 ligase complex and ubiquitination and degradation of targets^8,19^. To confirm the validity of this approach, we showed that FBXL3ΔF does not interact with SKP1 or CUL1, but maintains the ability to bind CRY1 and CRY2 (Fig. 1A). We used sequential immunoprecipitation (IP) to first purify FLAG-tagged FBXL3 (wildtype or ΔF), followed by purification of HA-tagged CRY1/2-containing complexes from the FLAG peptide eluate to focus only on proteins present in complexes containing both FBXL3 and either CRY1 or CRY2 (Fig. 1B). We included three control samples to exclude proteins associated non-specifically with the solid support or with FBXL3 independent of CRY1/2. This two-step, sequential IP greatly reduced the presence of non-specific partners in our final analysis, thus increasing confidence in identified targets (Fig. 1C).

We performed the experiments and analysis described above in triplicate (Supplementary Fig. 1) and used Integrated Proteomics Pipeline - IP2 (Integrated Proteomics Applications, Inc., San Diego, CA. http://www.integratedproteomics.com/) to identify >6000 proteins in all of our samples combined. For 141 of these proteins, we detected at least 30 spectral counts spread across at least five of the six experimental samples (those purified from cells expressing FLAG-FBXL3ΔF and either HA-CRY1 or HA-CRY2, Supplementary Table 1). We were surprised to find several mitochondrial proteins among those identified in our initial screen (Fig. 1D). We purified mitochondria from livers of *Cry1*^−/−^ and *Cry2*^−/−^ mice collected at zeitgeber time 16 (ZT16, sixteen hours after lights on) during the dark phase when CRY protein levels are high, but were unable to detect CRY1 or CRY2 in mitochondria (Supplementary Fig. 2). Therefore, we excluded mitochondrial and other membrane-associated proteins from further analysis. We focused on candidates for which at least 95% of the peptides detected in our experiments were found in the CRY-positive fraction (rather than negative control) samples (Fig. 1E). Furthermore, we used publicly available data in the so-called Contaminant Repository of Affinity Purification (CRAPome) to exclude from further consideration all proteins typically detected in more than 25% of negative control samples in APMS experiments using a similar setup^20^. These stringent filters left us with a short list of high confidence components of FBXL3- and CRY-containing complexes, including Tousled-like kinase TLK2 (Fig. 1F). We expect that the interactions of several of these proteins with FBXL3 and CRY1/2 are biologically meaningful. Here, we focus on TLK2 to demonstrate that CRY1 and CRY2 act as co-factors for SCF^FBXL3^ to target proteins involved in the DNA damage response and cell cycle, as an extension of our earlier work involving c-MYC^8^.

### TLK2 interacts with FBXL3 and CRY

**Figure 2:**
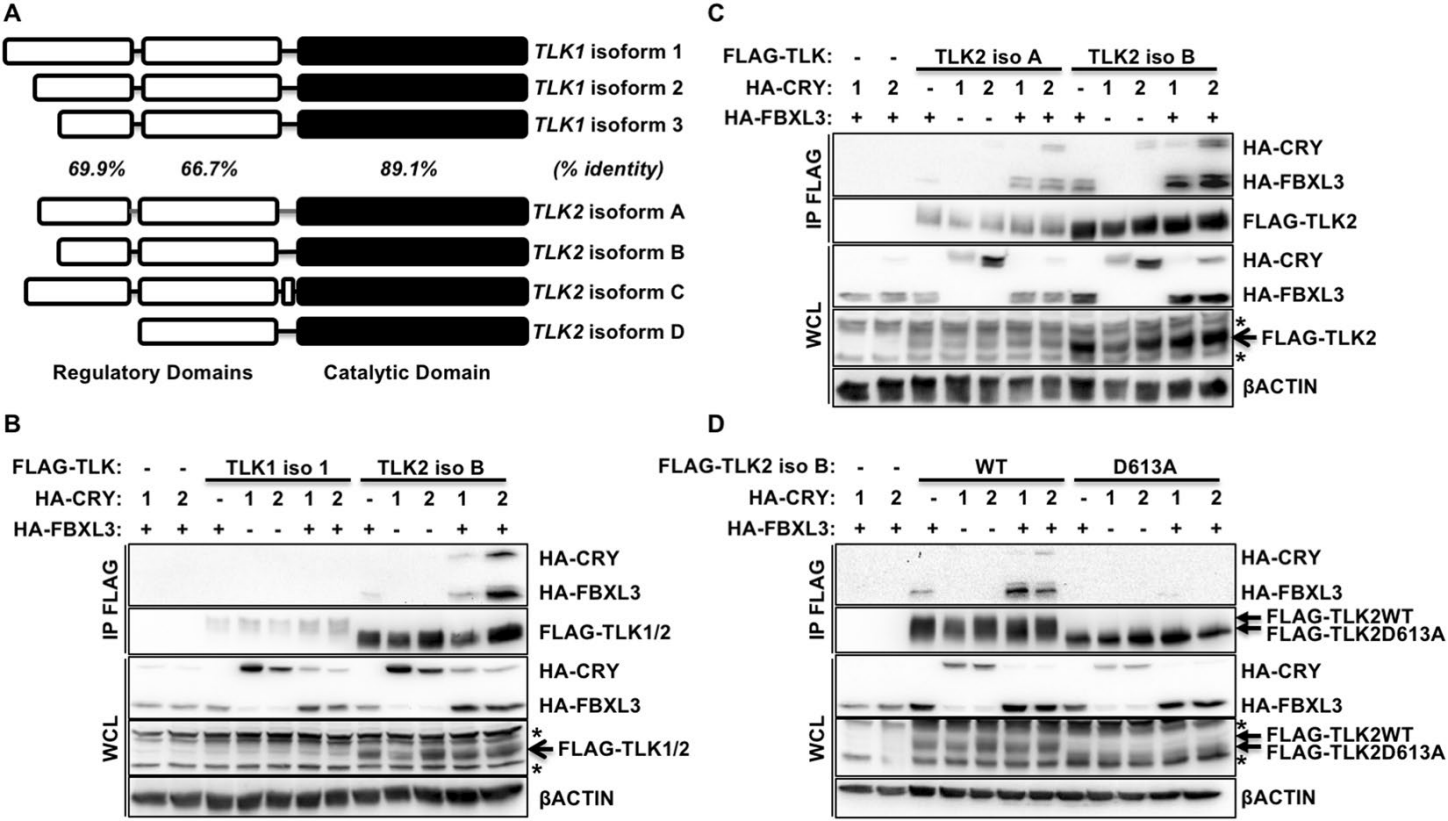
TLK2 interacts with FBXL3 and CRY. (**A**) TLK1 and TLK2 isoform homology in the indicated domains based on amino acid sequence. Percentages indicate amino acid identity of TLK1 and TLK2 in the indicated domains. (**B**–**D**) Detection of indicated proteins by IB in WCL or following FLAG IP from 293 T cells expressing the indicated plasmids. In (**D**) D613A is a catalytically inactive mutant of TLK2 iso B. * denotes non-specific signal.

TLK1 and TLK2 are highly homologous proteins (approximate overall homology is 80%) with the highest conservation at the C-terminus, which includes the catalytically active domain (Fig. 2A)^9^. TLK1 and TLK2 are each expressed from several transcripts, resulting in three or four unique protein isoforms, respectively (Fig. 2A). To assess whether TLK1 and/or TLK2 interact with FBXL3 and CRY1/2, we ectopically expressed epitope-tagged proteins in 293 T cells. While we were unable to express comparable amounts of FLAG-TLK1 and FLAG-TLK2, we did not detect interaction of overexpressed TLK1 with FBXL3 and/or CRY1 or CRY2 (Fig. 2B and Supplementary Fig. 3). Both the A and B isoforms of TLK2 interact with FBXL3, and this interaction is enhanced in the presence of CRY1 or CRY2 (Fig. 2C).

### Kinase activity of TLK2 is required for interaction with SCF^FBXL3+CRY^

Like many kinases, TLK2 is prone to autophosphorylation, in addition to its role in phosphorylating additional substrates^9^. We previously found that interaction of phosphorylated threonine 58 (pT58) in c-MYC with a phosphate binding loop (P loop) of CRY2 is critical for association of c-MYC with FBXL3 + CRY2^8^. Therefore, we examined whether the interaction with CRY and FBXL3 was affected by TLK2 kinase activity. Indeed, a catalytically inactive mutant of TLK2 (D613A) fails to associate with FBXL3 and CRY1 or CRY2 (Fig. 2D). These data indicate that TLK2 kinase activity is required for its interaction with FBXL3 and CRY. This may reflect a requirement for autophosphorylation of TLK2 or phosphorylation of another component of the complex by TLK2. Mutation of several individual serine or threonine residues in TLK2 did not prevent interaction with FBXL3 and CRYs (not shown).

### CRY and/or FBXL3 decrease TLK2 protein expression

**Figure 3:**
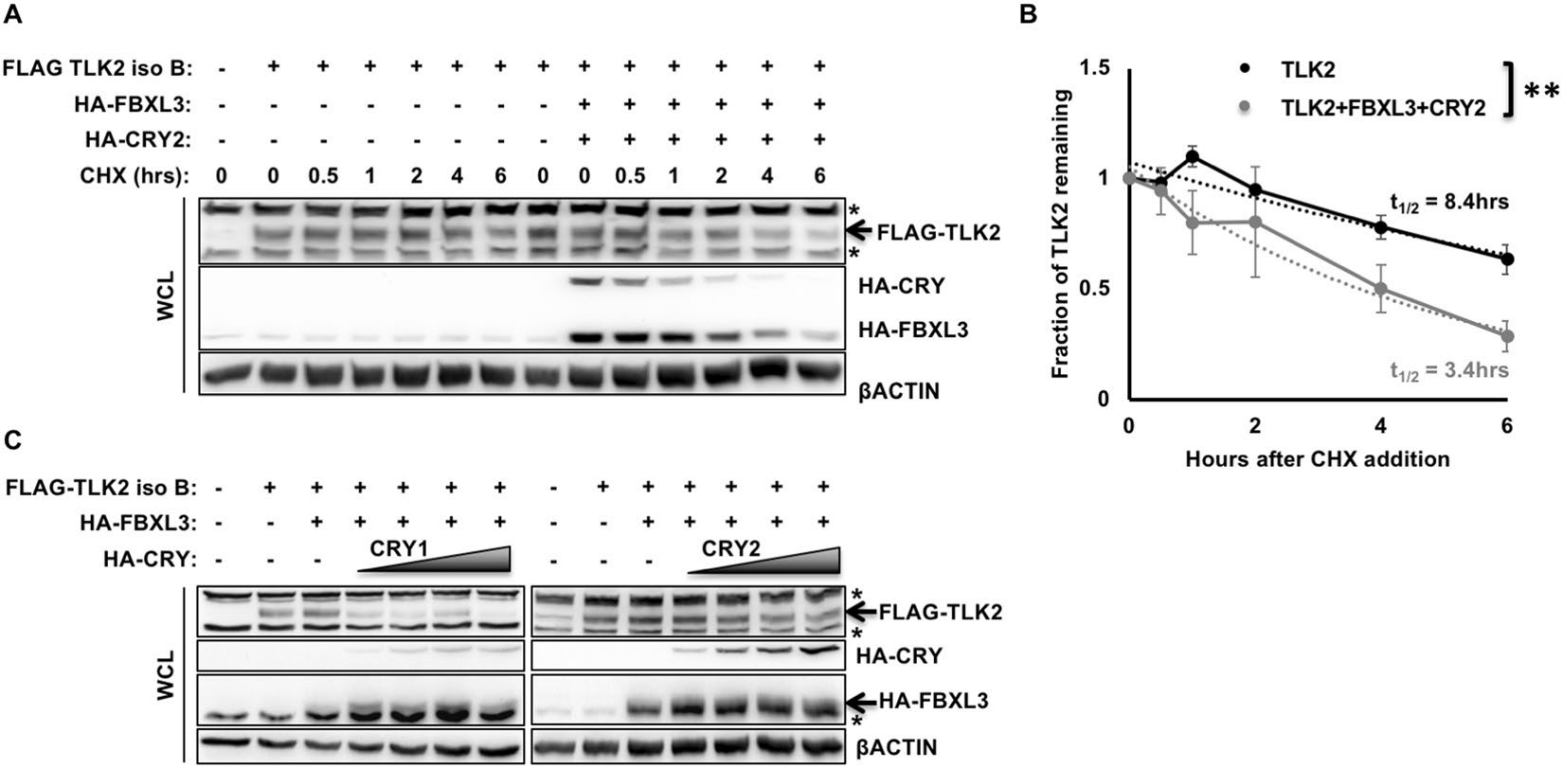
CRY1/2 and FBXL3 affect TLK2 stability. (**A,C**) Proteins detected by IB in WCL or following FLAG IP from 293 T cells expressing the indicated plasmids and following treatment with CHX for the indicated times. In (**C**) shaded triangles indicate increasing HA-CRY1 or HA-CRY2 expression. * denotes non-specific signal. (**B**) Quantitation of IB data from three experiments as shown in (**A**). Each data point represents the mean ± s.e.m. of the signal for FLAG-TLK2 overexpressed alone (black) or with overexpressed FBXL3 + CRY2 (gray) divided by the signal for βACTIN from the same sample, normalized to the sample collected before addition of CHX (t = 0). The dotted lines represent the best exponential fit to each data set, from which the half-lives were calculated according to the formula t_1/2_ = Tln(2), where T is derived from the exponential fit equation N(t) = N0exp(−t/T). **P < 0.01 by two-way ANOVA.

After determining that CRY and FBXL3 interact with TLK2, we wanted to determine whether they modulate TLK2 stability. First, we examined the effect of CRY and FBXL3 overexpression on turnover of overexpressed TLK2. In 293 T cells treated with cycloheximide, transient overexpression of CRY and FBXL3 increased TLK2 turnover (Fig. 3A,B). We also detected decreased TLK2 protein inversely correlated with the amount of CRY1 or CRY2 expressed in the presence of FBXL3 (Fig. 3C). Inhibition of the proteasome prevented the FBXL3 and CRY mediated decrease in FLAG-TLK2 protein levels (Supplementary Fig. 4).

### Endogenous CRYs influence TLK2 protein levels

**Figure 4:**
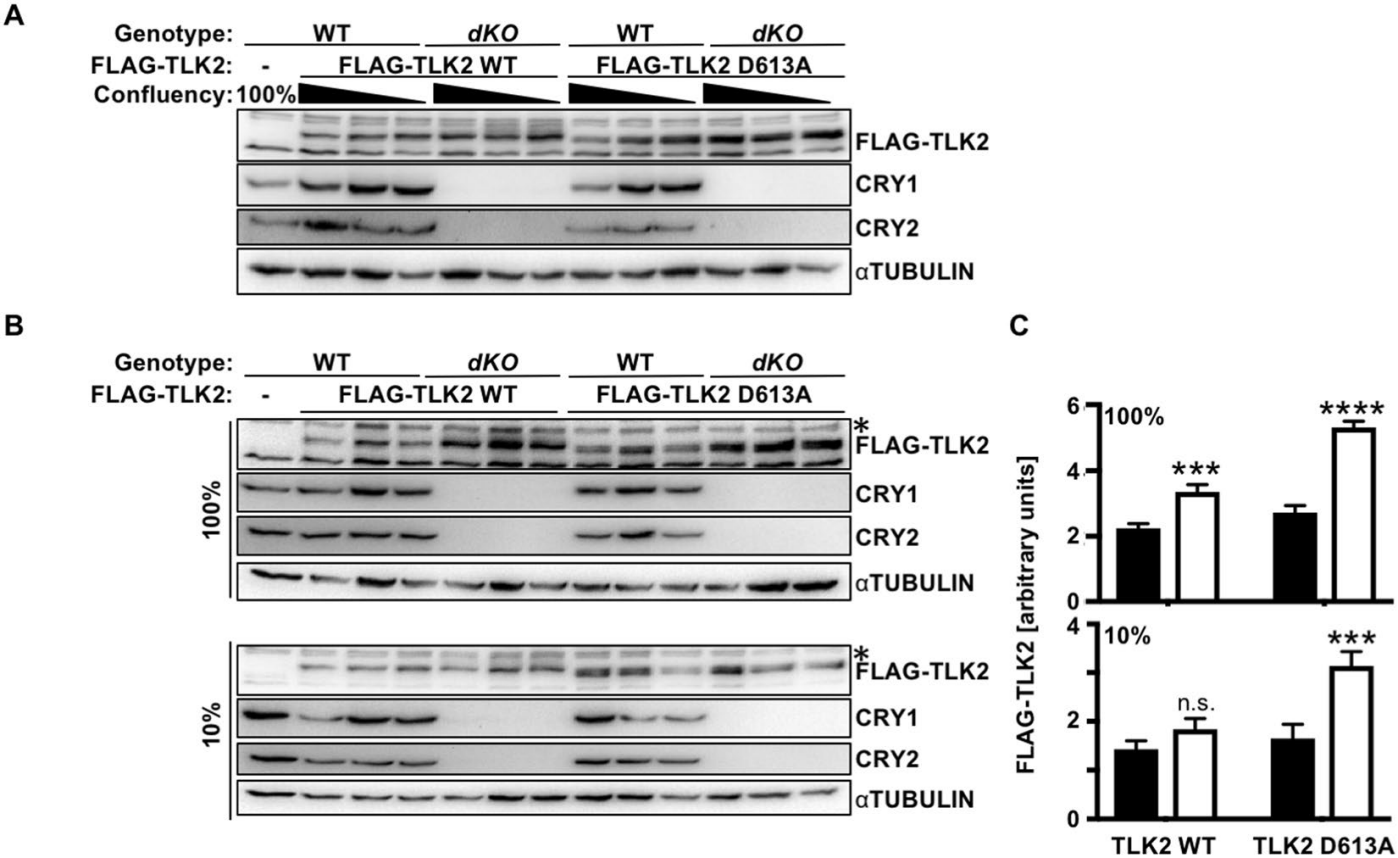
Endogenous CRY1 and CRY2 impact TLK2 protein expression. (**A,B**) Detection of indicated proteins in lysates prepared from mouse adult skin fibroblasts from *WT* and *Cry1*^−/−^;*Cry2*^−/−^ (*dKO*) mice stably expressing doxycycline-inducible FLAG-TLK2 WT or FLAG-TLK2D613A (KD) and at the plating confluencies as indicated. In (**A**), triangles indicate decreasing plating density (100%, 20%, 10%) 48 hours prior to lysis. (**C**) Quantitation of data shown in (**B**). Data represent mean ± s.d. for biological triplicates FLAG-TLK2 normalized to non-specific band (*) in lysates from wildtype (black bars) or *Cry1*^−/−^;*Cry2*^−/−^ (white bars) cells. P values were determined using two-way ANOVA followed by Sidak’s multiple comparison testing. ***^,^****P < 0.001, 0.0001 vs. WT cells.

To investigate the impact of endogenous CRY1 and CRY2 on TLK2, we stably expressed tetracycline inducible FLAG-tagged human TLK2 in adult mouse skin fibroblasts. While studying TLK2 in fibroblasts, we found that cell density altered the amount of TLK2 detected (Fig. 4A). Regardless, TLK2 protein levels are consistently elevated in *dKO* fibroblasts compared to wildtype cells (Fig. 4A–C and Supplementary Figs 5 and 6A). Deletion of either *Cry1* or *Cry2* also seems to enhance TLK2 but the effects were less clear (Supplementary Fig. 5). While overexpressed catalytically inactive TLK2 did not interact with overexpressed FBXL3 and CRY1/2, its expression was enhanced in *dKO* fibroblasts (Fig. 4A–C, and Supplementary Figs 5 and 6A). *Cry1/2* genotype had no effect on the expression of endogenous *Tlk2* mRNA (Supplementary Fig. 6B), but to our surprise doxycycline-induced expression of *FLAG-TLK2* mRNA was somewhat elevated in CRY-deficient cells compared to wildtype (Supplementary Fig. 6C). Together, these data support the conclusion that CRY1 or CRY2 can promote destabilization of TLK2 by recruiting it to SCF^FBXL3^, though enhanced transcription of the virally encoded exogenous TLK2 may contribute to the observed increase in FLAG-TLK2 protein detected in CRY-deficient cells as well.

## Discussion

Here we report the identification of substrates recruited to SCF^FBXL3^ by CRY1 and/or CRY2 in addition to the CRY2-dependent targeting of phosphorylated c-MYC that we previously reported^8^. While the APMS approach yielded many non-specific partners as others have reported^20^, we used stringent filters to focus on the most likely meaningful candidates, including the Tousled-like kinase TLK2.

In addition to TLK2, our data suggest several additional proteins as likely targets of SCF^FBXL3+CRY^-mediated ubiquitination and degradation. We identified the RNA editing enzyme ADAR as a potential target of FBXL3 + CRY-mediated ubiquitination (Fig. 1F). ADAR expression and RNA editing activity exhibit circadian oscillation in mouse livers^21^. In addition, ADAR regulates CRY2 expression by modulating microRNA targeting of its 3′ untranslated region (3′UTR), such that *Adarb1*^−/−^ mice express elevated levels of CRY2 protein^21^. Our data indicate that SCF^FBXL3+CRY^ may target ADAR for proteasomal degradation, suggesting another connection between circadian clocks and RNA editing. We also detected several ribosomal proteins as potential substrates for SCF^FBXL3+CRY^ ubiquitination (Fig. 1D,E). Recent findings have demonstrated that ribosome assembly and the translation of subsets of RNAs are regulated by circadian clocks^22–24^. SCF^FBXL3+CRY^-driven rhythms in turnover of ribosome components could contribute to such observations. The DNA Damage-Binding Protein 1, DDB1, is a component of CUL4-containing E3 ligase complexes and was recently shown to interact with and destabilize CRY1^25^. Its appearance in our screen may reflect FBXL3-independent CRY1 interaction or it could be a false positive given its relatively frequent occurrence in APMS screens performed in 293 T cells^20^.

TLK1 and TLK2 are regulated by the cell cycle and function in chromatin assembly and DNA damage response pathways^9,13,15,26^. Unlike c-MYC, which is specifically targeted by CRY2, TLK2 seems to be recruited to SCF^FBXL3^ by either CRY1 or CRY2. Similar to c-MYC, TLK2 interacts with FBXL3 and that interaction is enhanced in the presence of CRY. Since a catalytically inactive mutant of TLK2 (D613A) does not interact with FBXL3 + CRY, their interaction seems to depend on phosphorylation of one or more of the components. However, the stable expression of TLK2 D613A was enhanced in *Cry1*^−/−^*;Cry2*^−/−^ cells compared to wildtype cells, at least as much as that of wildtype TLK2 (Fig. 4). We suspect that this reflects phosphorylation of the relevant substrate by endogenous TLK2 or a related kinase but cannot exclude other possible explanations (Supplementary Fig. 7). Previous studies demonstrated that TLK2 kinase activity peaks when its protein level is at a minimum, during S phase^9^. Intriguingly, we observed an inverse correlation between cell density and exogenously expressed TLK2 protein levels. Interestingly, plating density had a stronger impact on the protein level of catalytically inactive TLK2 than on wildtype TLK2. We suspect that expression of exogenous wildtype TLK2 could mask the inactivation of endogenous TLK2 in confluent cells. The interactions between cell plating density, *Cry1/2* genotype, and TLK2 kinase activity in determining TLK2 protein levels suggest a complex interplay between these factors in the regulation of TLK2 degradation.

While we did not detect a strong preference of TLK2 for CRY1 or CRY2, FBXL3 and CRY may preferentially interact with TLK2 compared to TLK1. TLK1 and TLK2 are highly homologous proteins and both function in DNA replication by regulating chromatin assembly through phosphorylation of the ASF1 histone chaperone proteins^13^. Both TLK1 and TLK2 regulate cell cycle progression in undamaged cells, function in the DNA damage response, and modulate DNA replication. However, two unique findings distinguish them. First, TLK2 uniquely regulates checkpoint recovery after DNA damage during the G2 phase of the cell cycle. Also, TLK2 is frequently amplified in breast cancers^13,15–18,26–29^, while the same has not been observed for TLK1. Since TLK1 expression was much lower than that of TLK2 in our experiments, TLK1 may associate with SCF^FBXL3+CRY^ but at a level below our limit of detection. If SCF^FBXL3^ and CRY specifically regulate TLK2 and not TLK1, that could point to circadian regulation of checkpoint recovery in the cell cycle after DNA damage. Circadian rhythms are intimately intertwined with the cell cycle in diverse organisms^30–32^, and the mechanistic connection described here could contribute to their interrelationship.

The kinase activity and phosphorylation state of TLK2 change throughout the cell cycle^9^, though the mechanisms underlying this modulation are unknown. TLK2 activity is also suppressed in response to DNA damage by ATM-dependent signaling^29^. Intriguingly, FBXL3 is one of only four F-box proteins that exhibited significantly enhanced association with CUL1 after DNA damage in a recent study^33^, suggesting a critical role for SCF^FBXL3^ in response to DNA damage. Both c-MYC and TLK2 are degraded after DNA damage, and are targeted by SCF^FBXL3+CRY1/2^ complexes, which are expected to be more active in response to damage^33,34^. Further investigation will determine whether SCF^FBXL3+CRY1/2^ regulates TLK2, c-MYC, or other substrates during the cell cycle and/or in response to DNA damage. Also, the relationship between DNA damage signaling, TLK2 kinase activity, and SCF^FBXL3+CRY^-mediated ubiquitination is not yet clear. The SCF^FBXL3+CRY^-mediated post-translational regulation of TLK2 described here suggests another mechanistic link between clocks and cell cycle progression and timing.

## Methods

### Mass spectrometry

#### Reagents and chemicals

Deionized water (18.2 MΩ, Barnstead, Dubuque, IA) was used for all preparations. Buffer A consists of 5% acetonitrile 0.1% formic acid, buffer B consists of 80% acetonitrile 0.1% formic acid, and buffer C consists of 500 mM ammonium acetate and 5% acetonitrile.

#### Sample preparation

Proteins were precipitated in 23% TCA (Sigma-Aldrich, St. Louis, MO, Product number T-0699) at 4 °C O/N. After 30 min. centrifugation at 18000 × g, protein pellets were washed 2 times with 500 ul ice-cold acetone. Air-dried pellets were dissolved in 8 M urea/100 mM Tris pH 8.5. Proteins were reduced with 1 M Tris (2-carboxyethyl) phosphine hydrochloride (Sigma-Aldrich, St. Louis, MO, product C4706) and alkylated with 500 mM 2-Chloroacetamide (Sigma-Aldrich, St. Louis, MO, product 22790-250G-F). Proteins were digested for 18 hrs. at 37 °C in 2 M urea, 100 mM Tris pH 8.5, 1 mM CaCl_2_ with 2 ug trypsin (Promega, Madison, WI, product V5111). Digest was stopped with formic acid, 5% final concentration. Debris was removed by centrifugation, 30 min 18000 × g.

#### MudPIT microcolumn

A MudPIT microcolumn^35^ was prepared by first creating a Kasil frit at one end of an undeactivated 250 μm ID/360 μm OD capillary (Agilent Technologies, Inc., Santa Clara, CA). The Kasil frit was prepared by briefly dipping a 20–30 cm capillary in well-mixed 300 μL Kasil 1624 (PQ Corporation, Malvern, PA) and 100 μL formamide, curing at 100 °C for 4 hrs, and cutting the frit to ~2 mm in length. Strong cation exchange particles (SCX Partisphere, 5 μm dia., 125 Å pores, Phenomenex, Torrance, CA) was packed in-house from particle slurries in methanol 2.5 cm. An additional 2.5 cm reversed phase particles (C18 Aqua, 3 µm dia., 125 Å pores, Phenomenex) were then similarly packed into the capillary using the same method as SCX loading, to create a biphasic column. An analytical RPLC column was generated by pulling a 100 μm ID/360 μm OD capillary (Polymicro Technologies, Inc, Phoenix, AZ) to 5 μm ID tip. Reversed phase particles (Aqua C18, 3 μm dia., 125 Å pores, Phenomenex, Torrance, CA) were packed directly into the pulled column at 800 psi until 12 cm long. The MudPIT microcolumn was connected to an analytical column using a zero-dead volume union (Upchurch Scientific (IDEX Health & Science), P-720-01, Oak Harbor, WA). LC-MS/MS analysis was performed using an Agilent Technologies 1200 HPLC pump and a Thermo Orbitrap Velos Pro using an in-house built electrospray stage. MudPIT experiments were performed with steps of 0% buffer C, 30% buffer C, 70% buffer C, 100% C, and 90/10% buffer C/B, being run for 3 min at the beginning of each gradient of buffer B. Electrospray was performed directly from the analytical column by applying the ESI voltage at a tee (150 μm ID, Upchurch Scientific)^35^. Electrospray directly from the LC column was done at 2.5 kV with an inlet capillary temperature of 325 °C. Data-dependent acquisition of MS/MS spectra with the Orbitrap Velos Pro were performed with the following settings: MS/MS on the 10 most intense ions per precursor scan; 1 microscan; reject unassigned charge state and charge state 1; dynamic exclusion repeat count, 1; repeat duration, 30 second; exclusion list size 300; and exclusion duration, 20 second.

#### Data analysis

Protein and peptide identification and protein quantitation were done with Integrated Proteomics Pipeline - IP2 (Integrated Proteomics Applications, Inc., San Diego, CA. http://www.integratedproteomics.com/). Tandem mass spectra were extracted from raw files using RawConverter^36^ and were searched against a Uniprot human protein database with fusion proteins and reversed sequences using ProLuCID^37,38^. The search space included all fully-tryptic and half-tryptic peptide candidates with a fixed modification of 57.02146 on cysteine. Peptide candidates were filtered using DTASelect^39^.

### Sequential immunoprecipitation

Samples were prepared for mass spectrometry analysis following the sequential immunoprecipitation protocol previously outlined^8^. After the anti-HA beads were then spun down and washed 3X with lysis buffer containing 1%TX-100, 50 mM HEPES (pH 7.4), 138 mM KCl, 4 mM NaCl, 50 mM sodium pyrophosphate, 100 mM sodium fluoride, 10 mM EDTA, 1 mM EGTA pH 8, 50 μM PMSF, 1 mM β-Glycerophosphate, 1 mM sodium orthovanadate, and protease inhibitors (Roche cat # 11697498001) similar to previously described^40^, they were incubated in 150uL HA peptide (Sigma cat# I2149), diluted 1:50 in PBS, for 1.5 hours at 4 °C while nutating. 90% of the eluates were flash frozen for subsequent mass spectrometry analysis, while 10% were boiled in SDS sample buffer, run on an SDS-PAGE gel and stained using a silver stain kit (Thermo Scientific Pierce Biotech cat# P124612).

### Plasmids

pcDNA3-HA-CRY2, pcDNA3-HA-FBXL3, pcDNA3-MYC-CRY2, pcDNA3-2xFLAG-FBXL3, and pcDNA3-2xFLAG-FBXL3ΔF are as described previously^8^. *Cry1* and *Fbxl3ΔF* coding sequences were transferred to pcDNA3.1-based HA-epitope or pcDNA3.1-based MYC-epitope vectors using standard protocols. cDNA encoding human *Tlk2* isoform A was generated by RT-PCR from RNA extracted from 293 T cells. Plasmids expressing TLK2 isoform A were made by cloning the *Tlk2* isoform A cDNA into a pcDNA3.1-based V5-epitope tagged vector using standard protocols. pcDNA3-2xFLAG-TLK2 isoform A and pcDNA3-2xFLAG-TLK2 isoform B were generated using Q5 Site-Directed Mutagenesis (New England Biolabs Inc. cat # E0554S). pDONR223-TLK1, originally generated by was a gift from William Hahn & David Root (Addgene plasmid # 23727)^41^ and was cloned into a pcDNA3.1-based V5-epitope tagged vector using the pcDNA Gateway Directional TOPO Expression Kit (Invitrogen #K2440-20). pcDNA3.1-2xFLAG-TLK1 was then similarly generated using Q5 Site-Directed Mutagenesis. psPAX plasmid (Addgene plasmid 12260) and pMD2.G plasmid (Addgene plasmid 12259) deposited by Dr. Didier Trono, and used for infection, were also purchased from Addgene. pCW-Cas9 was a gift from Eric Lander & David Sabatini (Addgene plasmid # 50661)^42^ and the *Cas9* sequence was replaced with *Tlk2* cDNA using the Gibson Assembly Ultra Kit (Synthetic Genomics Inc. cat # GA1200-10). pCW-2xFLAG-TLK2 was then generated by introducing a 2xFLAG sequence using Q5 Site-Directed Mutagenesis. Plasmids containing TLK2 KD mutant was generated by mutating TLK2 WT to TLK2 D613A using Q5 Site-Directed Mutagenesis.

### Cell culture and transfection

All cells were grown in complete Dulbecco’s Modified Eagle Medium (DMEM) (cat #10569; Invitrogen) unless otherwise indicated. 293 T cell media was supplemented with 10% fetal bovine serum, and 1% penicillin and streptomycin. 293 T cells were grown in a 37 °C incubator maintained at 5% CO_2_ and 20% O_2_ (high oxygen). Adult skin fibroblasts were prepared from ear biopsies of adult mice of the indicated genotypes, as described previously^34^. Adult skin fibroblasts (ASFs) were used as primary (passaged no more than 10 times and grown in 3% oxygen). ASF media were supplemented with 15% fetal bovine serum, and 1% penicillin and streptomycin. ASFs were grown in a 37 °C incubator maintained at 5% CO_2_ and 3% O_2_ (low oxygen). Transfections for mass spectrometry analysis were carried out using calcium phosphate, while all other transfections were performed using polyethylenimine (cat #23966-2; PEI; Polysciences Inc, Warrington, PA) by standard protocols. For calcium phosphate transfections of a 10 cm plate of 293 T cells: 1–10 μg DNA was mixed with 450 μL sterile water, 150 μL 1 M CaCl_2_ was added, and the tube contents mixed by vortex. 600 μL 2xHBS pH 7.02 (140 mM NaCl, 1.5 mM Na_2_HPO_4_, 50 mM HEPES) was pipetted into a separate tube, and the DNA/CaCl_2_ mix was added dropwise to the 2xHBS and the contents of the tube were mixed by forcing air bubbles through the solution. For PEI transfections, 1–10 µg DNA was added to 1 mL serum free DMEM and mixed with 20 µl PEI. Solutions were mixed by vortex and incubated for 20 mins. For both transfection methods, the transfection solution was added dropwise to each plate after the 293 T cell media was changed. After 24 hours, the 293 T cell media was again replaced and protein extracts were isolated 48 hours post transfection. Cycloheximide (CHX) (Fisher cat # 50255724) was used at a concentration of 100 μg/ml as indicated.

### Generation of viruses and stable cell lines

Lentiviral pCW-2xFLAG-TLK2 was produced by transient transfection into HEK 293 T cells using psPAX and pMD2.G packaging plasmids for virus generation. Lentiviral supernatants were harvested 48 hours after transfection, filtered through a 0.45 μm filter, supplemented with 6 μg/ml polybrene (Sigma) and added to parental cell lines. The medium was replaced 24 hours after the virus and polybrene were added to the parental cells. 48 hours after viral transduction, the infected cells were split into selection media containing 2 μg/ml of puromycin (Sigma cat # P9620-10ML). Selection media were replaced every 2–3 days until selection was complete (2 days–1 week).

### Doxycycline induction of stable cell lines

ASF cells of the indicated genotypes stably expressing pCW-2xFLAG-TLK2 WT and pCW-2xFLAG-TLK2 KD were treated with 1 μM doxycycline (Sigma cat # D9891) for 48 hours to induce 2xFLAG-TLK2 expression.

### Immunoprecipitation and western blotting

293 T whole cell extracts were prepared using lysis buffer containing 1%TX-100, 50 mM HEPES (pH 7.4), 138 mM KCl, 4 mM NaCl, 50 mM sodium pyrophosphate, 100 mM sodium fluoride, 10 mM EDTA, 1 mM EGTA pH 8, 50 μM PMSF, 1 mM β-Glycerophosphate, 1 mM sodium orthovanadate, and protease inhibitors (Roche cat # 11697498001 or Thermo Scientific cat # 88265) as previously described (Lamia *et al*.^40^). ASF cell extracts were prepared from RIPA buffer containing 1% TX-100, 147 mM NaCl, 12 mM sodium deoxycholate, 0.1% SDS, 50 mM Tris pH 8.0, 10 mM EDTA, 50 μM PMSF, 1 mM β-Glycerophosphate, 1 mM sodium orthovanadate, 1 mM sodium fluoride, and protease inhibitors (Thermo Scientific cat # 88265). Antibodies used for immunoprecipitation were anti-FLAG M2 agarose beads (Sigma cat # A2220) and monoclonal anti-HA agarose beads (Sigma cat # A2095). Antibodies for Western blot were anti-FLAG polyclonal (Sigma cat # F7425), anti-HA polyclonal (Sigma cat # H6908), anti-MYC polyclonal (Sigma cat # C3956), anti-SKP1 (BD Biosciences cat # BDB610530), anti-CUL1 (Life Technologies cat # 71–8700), anti-αTubulin (Sigma cat # T5168), anti-βactin (Sigma cat # A1978), anti-AFG3L2[N1N2] (GeneTex cat # GTX102036), and CRY1-CT and CRY2-CT as described (Lamia *et al*., 2011).

### Mitochondrial fractionation

Mice were sacrificed after two weeks of entrainment to 12:12 LD cycles. The right lobe of each liver was extracted from the mice at the indicated zeitgeber times (ZT, hours after lights on), and washed 3X in cold PBS. All steps were done on ice or at 4 °C. 1 mL of mitochondrial lysis buffer containing 220 mM sorbitol, 70 mM sucrose, 50 mM MOPS pH 7.4, 5 mM EGTA and protease inhibitors (cat #11697498001; Roche, Switzerland), was added to each liver sample. The samples were then homogenized and samples centrifuged at 1000 × *g* for 10 minutes at 4 °C to pellet nuclei and debris. The supernatant was collected as the post-nuclear lysate. The sample was then centrifuged at 9500 × *g* for 10 minutes at 4 °C to pellet mitochondria. The supernatant (cytosolic fraction) was removed, and the mitochondrial pellet was resuspended in mitochondria wash buffer containing 220 mM sorbitol, 70 mM sucrose, 50 mM MOPS pH 7.4 and centrifuged again at 9500 × *g* for 10 minutes at 4 °C. This wash step was then repeated twice more. Finally, TX-100 was added to all samples at a final concentration of 1%, and samples were incubated on ice for 10 mins. Mitochondrial lysates were boiled in SDS sample buffer and 60 μg per sample were run on SDS-PAGE.

### Measurement of gene expression

RNA was extracted from adult mouse ear fibroblasts with Qiazol reagent using standard protocols (Qiagen cat # 799306). cDNA was prepared using iScript RT Supermix for RT-qPCR (Bio-Rad cat # 1708841) and analyzed for gene expression using quantitative real-time PCR with iQ SYBR Green Supermix (Biorad cat # 1708885). Primers used for qPCR were:

**Table:** 

	**Forward (5′-3′)**	**Reverse (5′-3′)**
*mTlk2*	ACTAGCGCAAAGGAACTCAATGTG	CCAGACCAGGCAAGACTCAGAC
*hTLK2*	GACGATGACGATAAAATGATGG	CACTCAAGGATCCGACGC
*mU36b4*	AGATGCAGCAGATCCGCA	GTTCTTGCCCATCAGCACC

### Mice

*Cry1*^−/−^*;Cry2*^−/−^ mice were from Dr. Aziz Sancar^43^. All animal care and treatments were in accordance with The Scripps Research Institute guidelines and regulations for the care and use of animals. All procedures involving experimental animals were approved by The Scripps Research Institute Institutional Animal Care and Use Committee (IACUC) under protocol #10-0019.

The mass spectrometry proteomics data have been deposited to the ProteomeXchange Consortium via the PRIDE [1] partner repository with the dataset identifier PXD009522 and 10.6019/PXD009522.

## Electronic supplementary material

Supplementary Information

Supplementary Table 1

## Data Availability

All data generated or analyzed during this study are either included in this published article (and its Supplementary Information files) or are available from the corresponding author on reasonable request.
