# Supplementary material for: The circadian E3 ligase complex SCFFBXL3+CRY targets TLK2
Source: Sci Rep. 2019 Jan 17;9:198. doi: 10.1038/s41598-018-36618-3 (PMC6336870; doi:10.1038/s41598-018-36618-3)
Supplement: Supplementary file 1 — Supplementary Information [file 41598_2018_36618_MOESM1_ESM.pdf]

Supplementary Material Related to

Title: The circadian E3 ligase complex $SCF^{FBXL3+CRY}$ targets TLK2

Authors: Stephanie Papp Correia[1], Alanna B. Chan[1], Megan Vaughan, Norjin Zolboot, Valerie Perea, Anne-Laure Huber, Anna Kriebs, James J. Moresco, John R. Yates III, and Katja A. Lamia[*]

Department of Molecular Medicine, The Scripps Research Institute, 10550 North Torrey Pines Road, La Jolla, CA 92037, USA

[1] These authors contributed equally to this work.

*Correspondence: klamia@scripps.edu

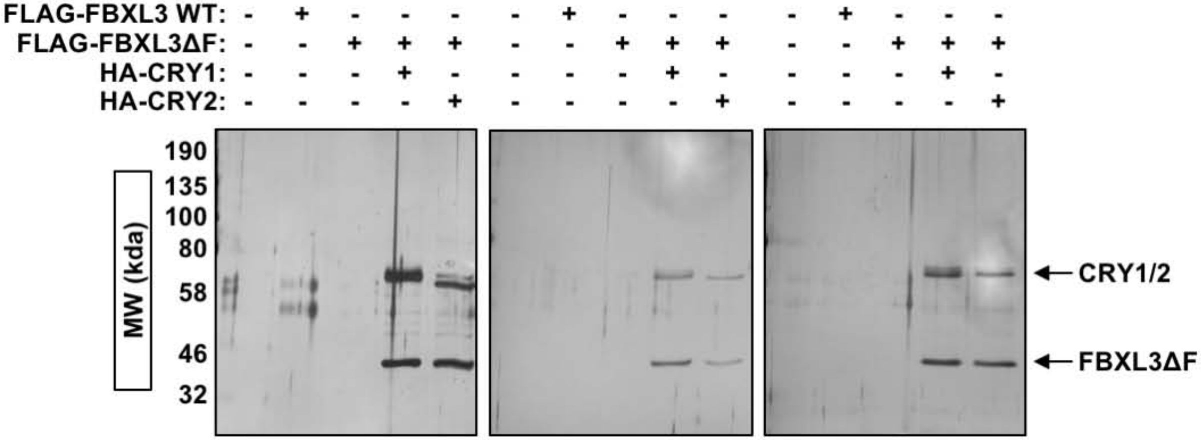

Supplementary Figure 1: APMS experiments performed in triplicate for screen. Related to Figure 1. Proteins detected by silver stain in samples prepared as shown in (Figure 1A) from 293T cells and separated by SDS-PAGE.

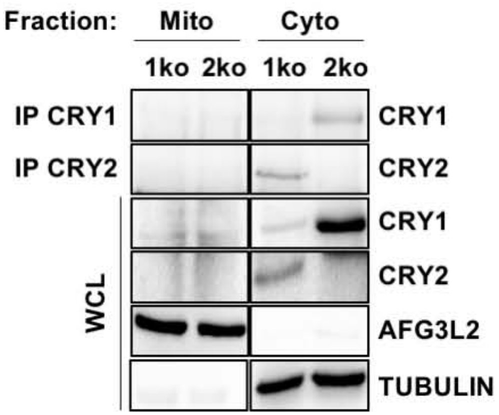

Supplementary Figure 2: No detectable CRY1 or CRY2 in liver mitochondria. Proteins detected by IB in protein samples or after CRY1 or CRY2 IP from mitochondrial (Mito) or cytoplasmic (Cyto) fractions prepared from *Cry1*$^{-/-}$ (1ko) or *Cry2*$^{-/-}$ (2ko) mice at zeitgeber time (ZT, hours after lights on) 16.

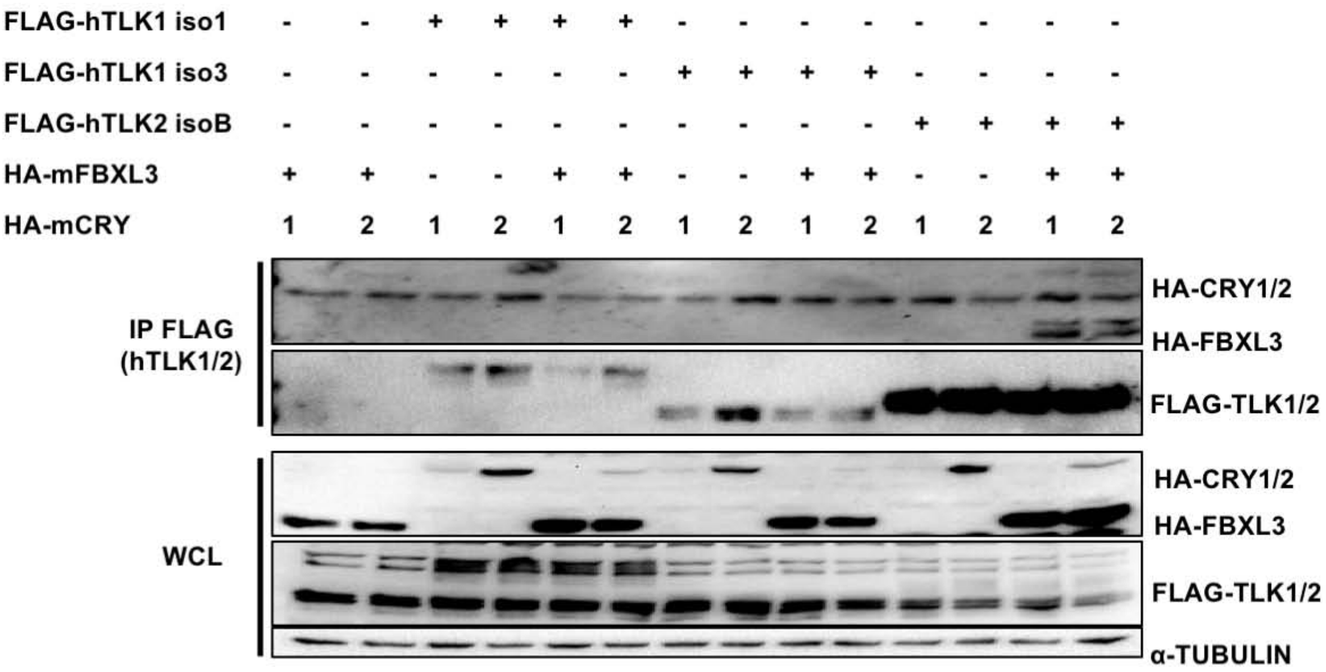

Supplementary Figure 3: TLK2 interacts with FBXL3 and CRY. Detection of indicated proteins by IB in WCL or following FLAG IP from 293T cells expressing the indicated plasmids

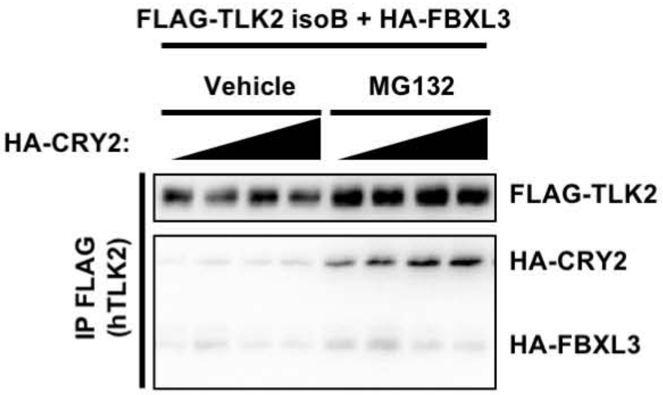

Supplementary Figure 4: FBXL3+CRY2 stimulate proteasomal degradation of TLK2
Proteins detected by IB after FLAG IP from 293T cells expressing the indicated plasmids.

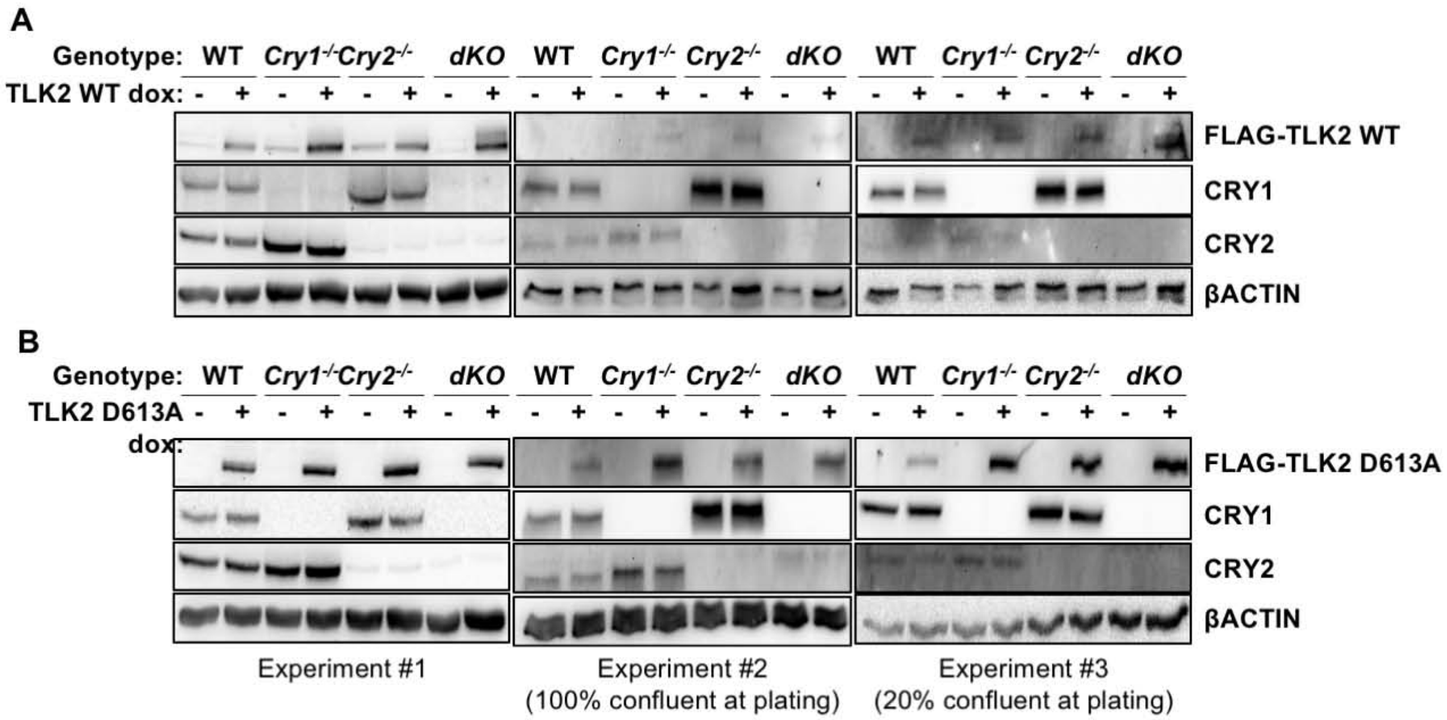

Supplementary Figure 5: Deletion of CRY1 or CRY2 increases TLK2 protein. Proteins detected by IB in WCL from mouse skin fibroblasts of the indicated genotypes stably expressing doxycycline-inducible wildtype (A) or catalytically inactive (B) FLAG-TLK2 following treatment with vehicle (DMSO) or doxycycline for 48 hours.

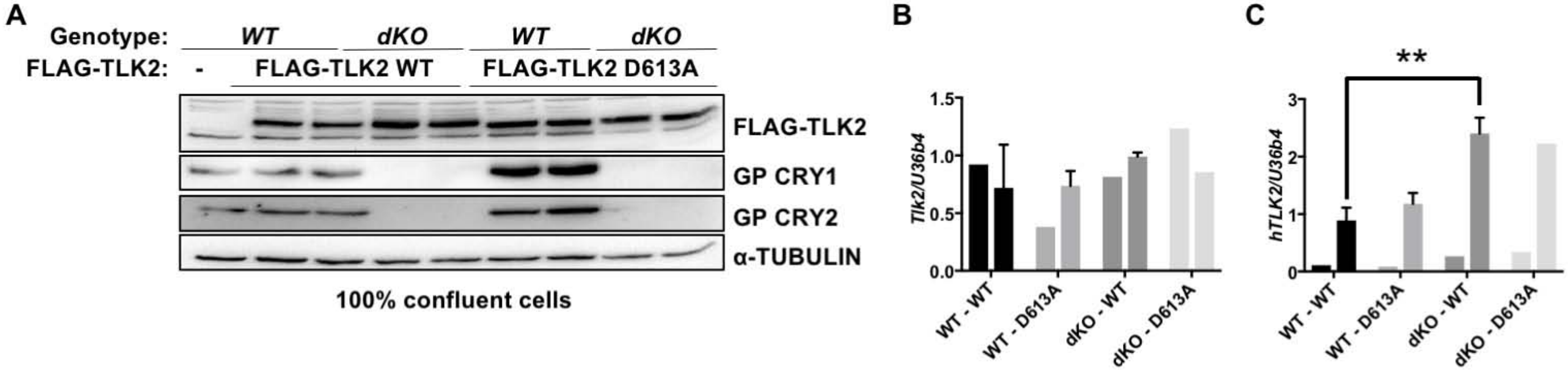

Supplementary Figure 6: Deletion of CRY1 or CRY2 increases TLK2 protein. (A) Proteins detected by IB in WCL from mouse skin fibroblasts of the indicated genotypes stably expressing doxycycline-inducible wildtype (WT) or catalytically inactive (D613A) FLAG-TLK2 following treatment with vehicle (DMSO) or doxycycline for 48 hours. (B,C) mRNA expression of endogenous mouse *Tlk2* (B) or exogenous human *TLK2* (C) normalized to mouse *U36b4* measured by quantitative real-time PCR in RNA prepared from cells plated in parallel to those examined in (A).

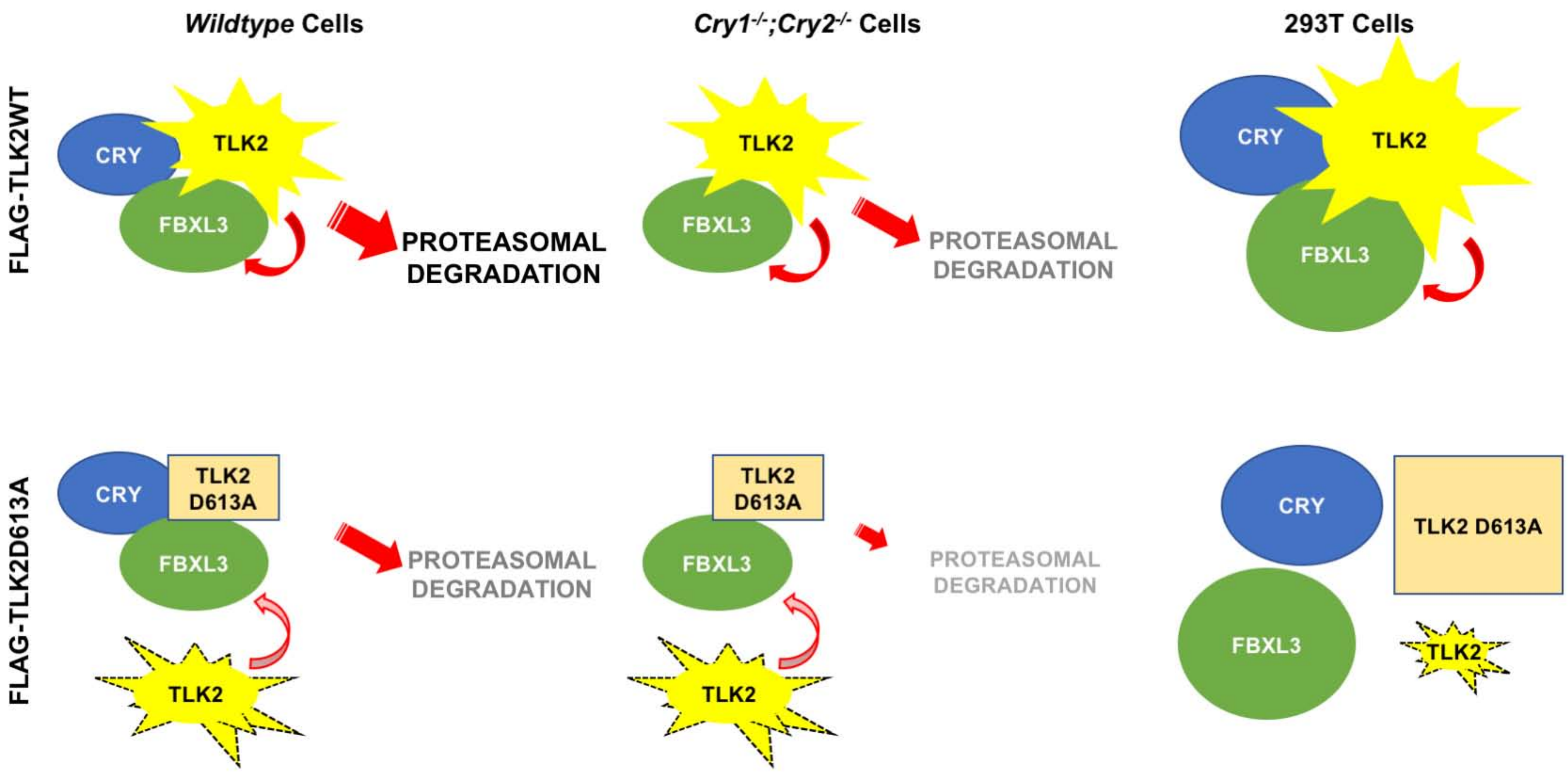

Supplementary Figure 7: Hypothetical model for FBXL3-CRY regulation of TLK2. Our data indicate that $SCF^{FBXL3+CRY1/2}$ contributes to TLK2 destabilization by promoting its ubiquitination. We propose that TLK2 enhances its own ubiquitylation and degradation by phosphorylating a component of the $SCF^{FBXL3+CRY}$ complex. In CRY-deficient cells, wildtype TLK2 may interact with FBXL3 to a lesser extent than when CRY1/2 are also present. (As seen in figure 2, TLK2 can interact with FBXL3 in the absence of CRY overexpression). In wildtype cells, we hypothesize that phosphorylation of the endogenous $SCF^{FBXL3+CRY1/2}$ complex by endogenous TLK2 enables its interaction with exogenous FLAG-TLK2D613A. This interaction would be weaker in CRY-deficient cells. The stronger impact of the D613A mutation on interaction with FBXL3 and CRY in 293T cells may reflect the fact that all three proteins are overexpressed, resulting in a much greater ratio of CRY and FBXL3 to endogenous TLK2 than in the experiments performed in primary fibroblasts.

FLAG IPs:

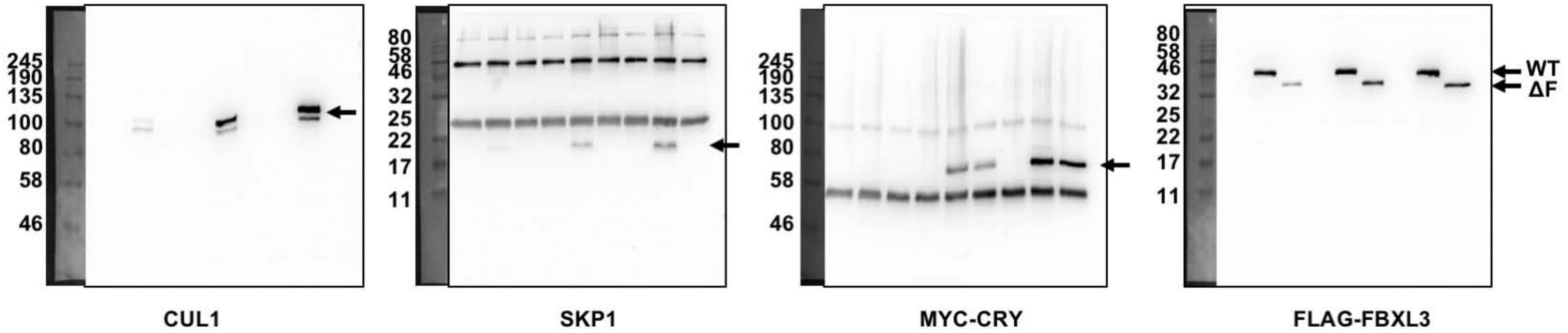

Lysates:

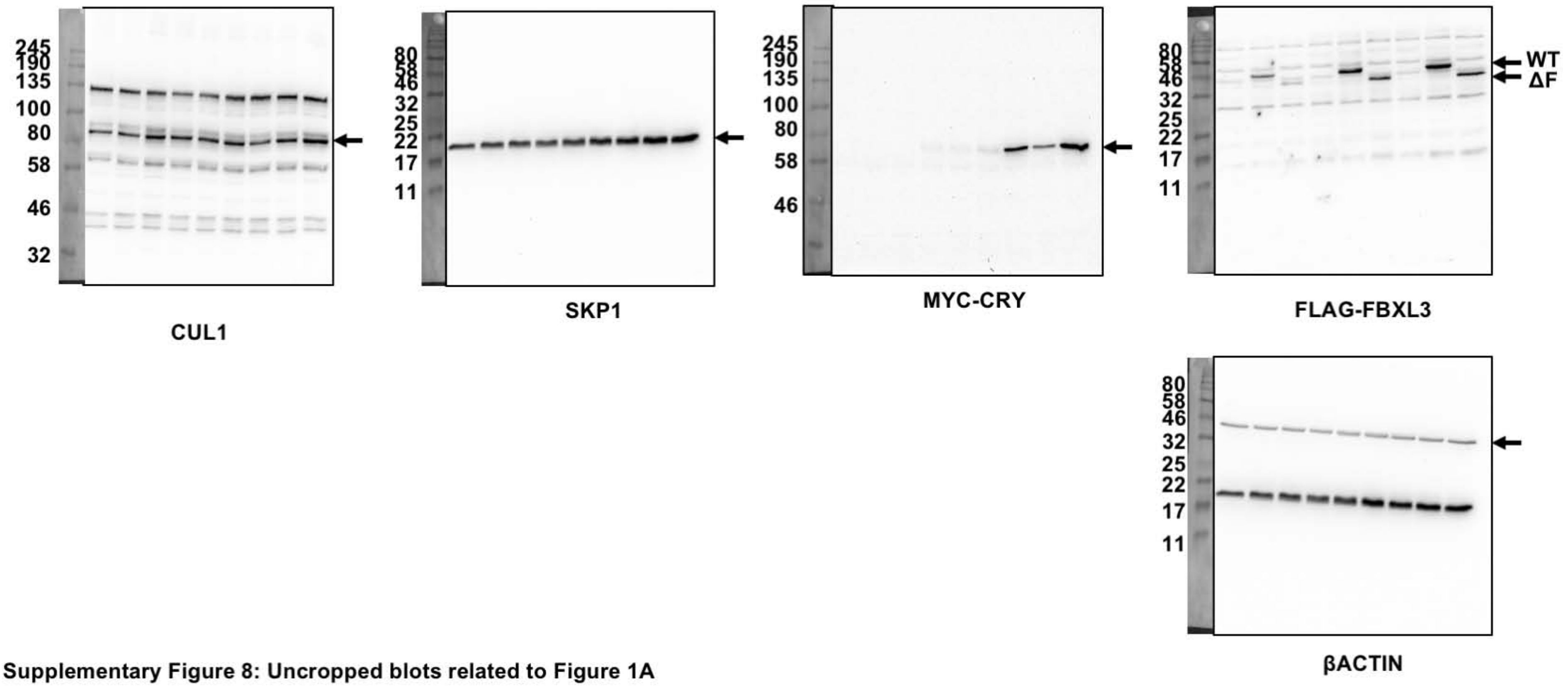

Supplementary Figure 8: Uncropped blots related to Figure 1A

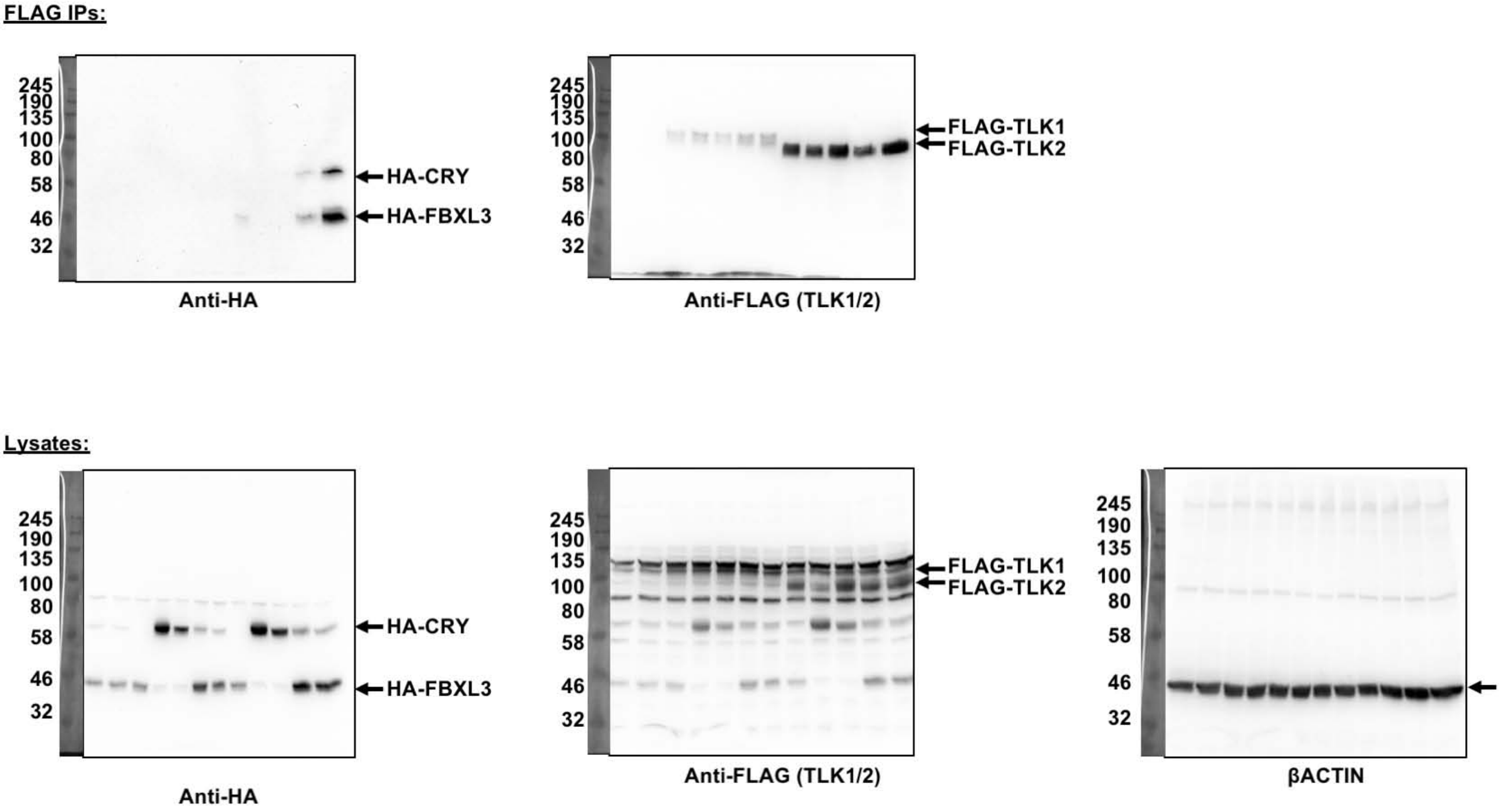

Supplementary Figure 9: Uncropped blots related to Figure 2B

FLAG IPs:

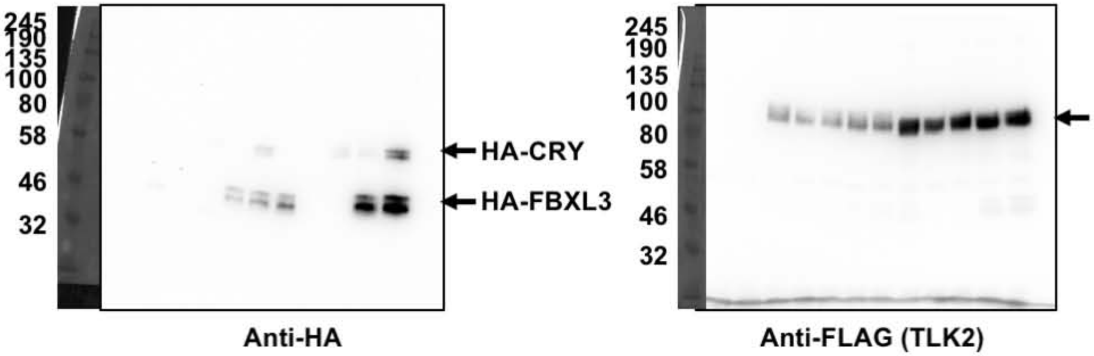

Lysates:

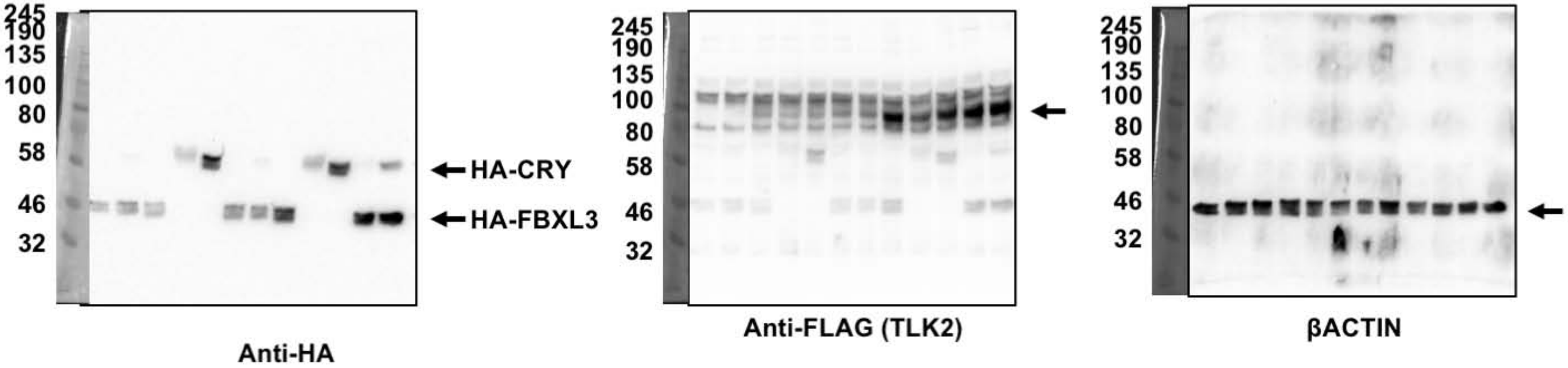

Supplementary Figure 10: Uncropped blots related to Figure 2C

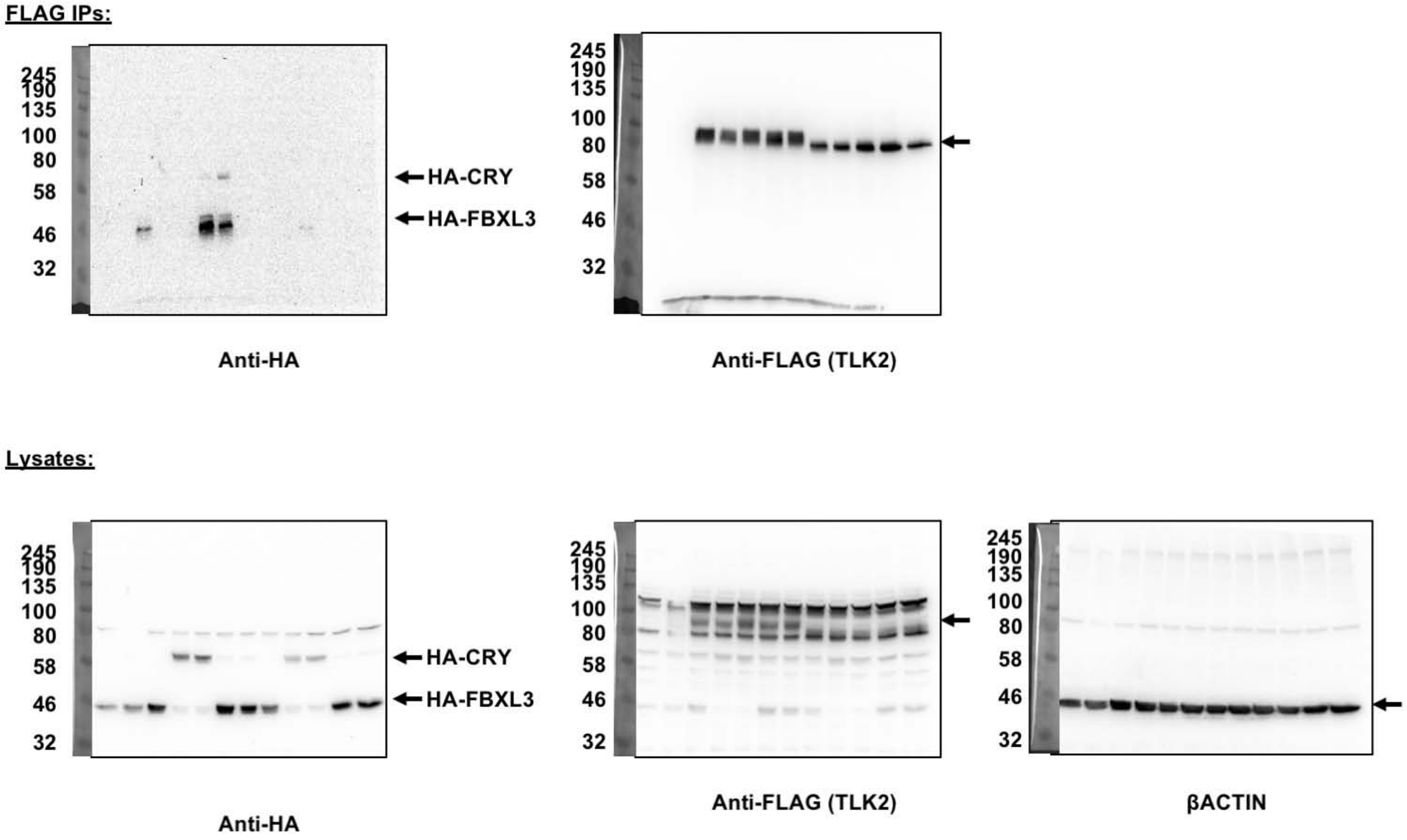

Supplementary Figure 11: Uncropped blots related to Figure 2D

Figure 3A (experiment 1):

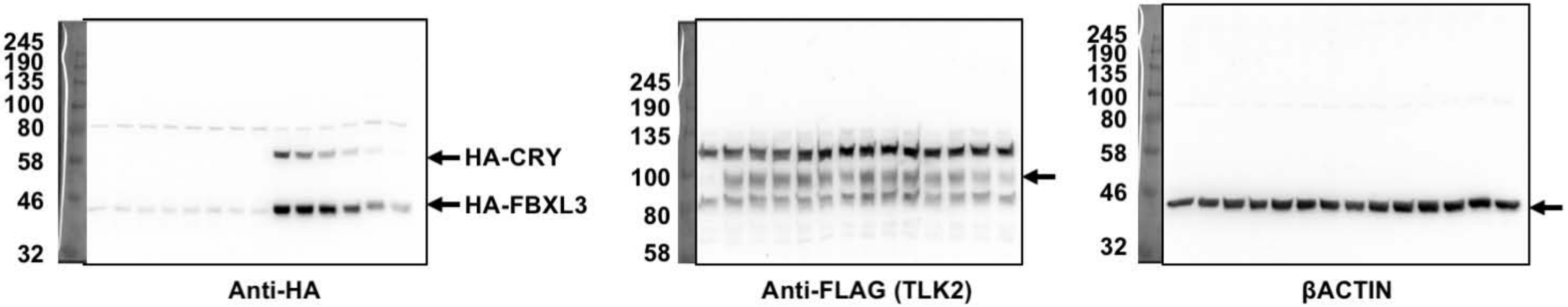

Figure 3B (experiment 2):

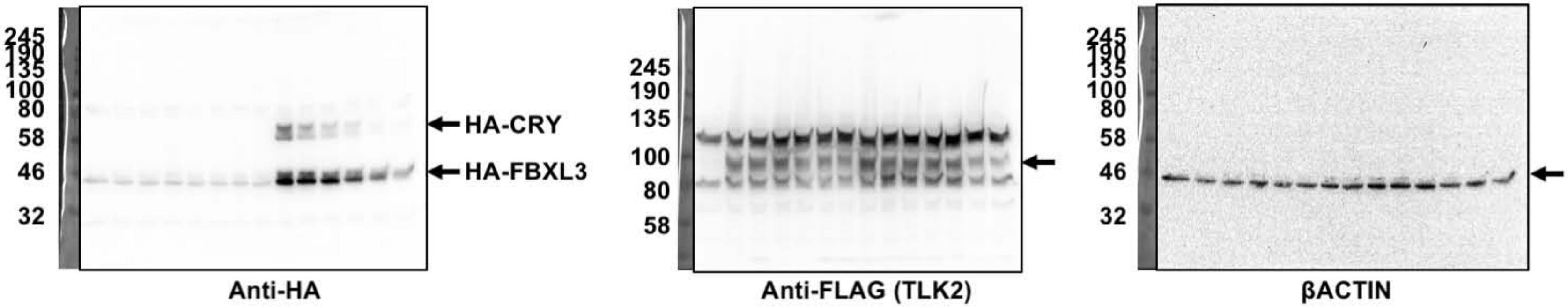

Figure 3B (experiment 3):

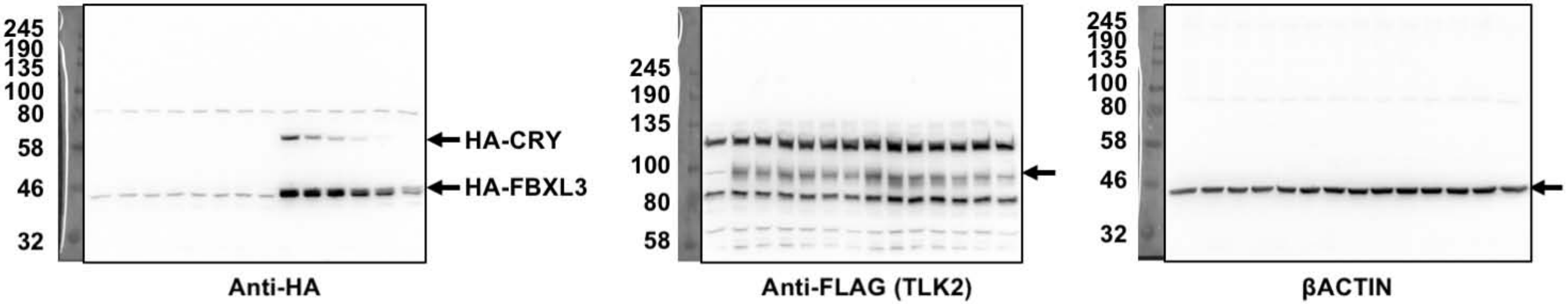

Supplementary Figure 12: Uncropped blots related to Figures 3A and 3B

Left Panels (CRY1):

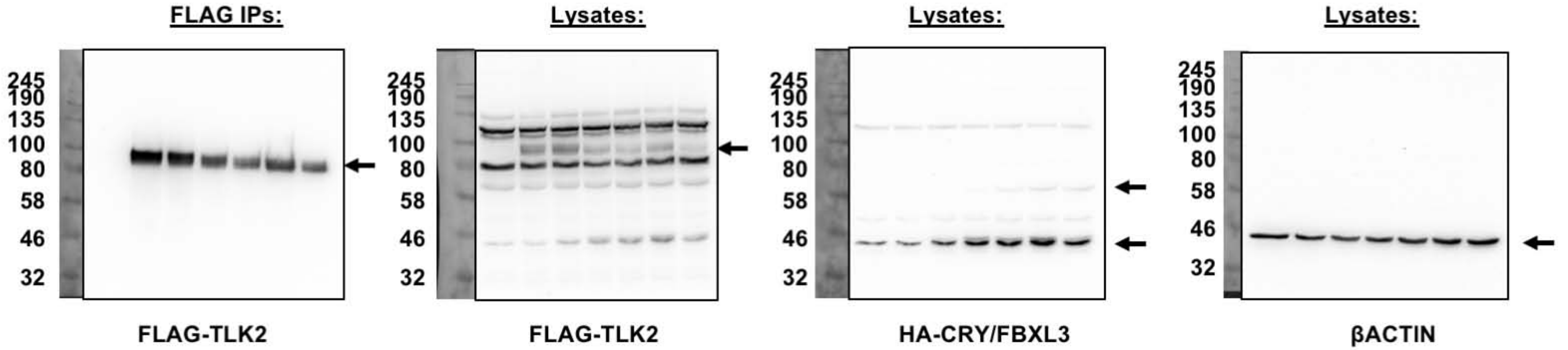

Right Panels (CRY2):

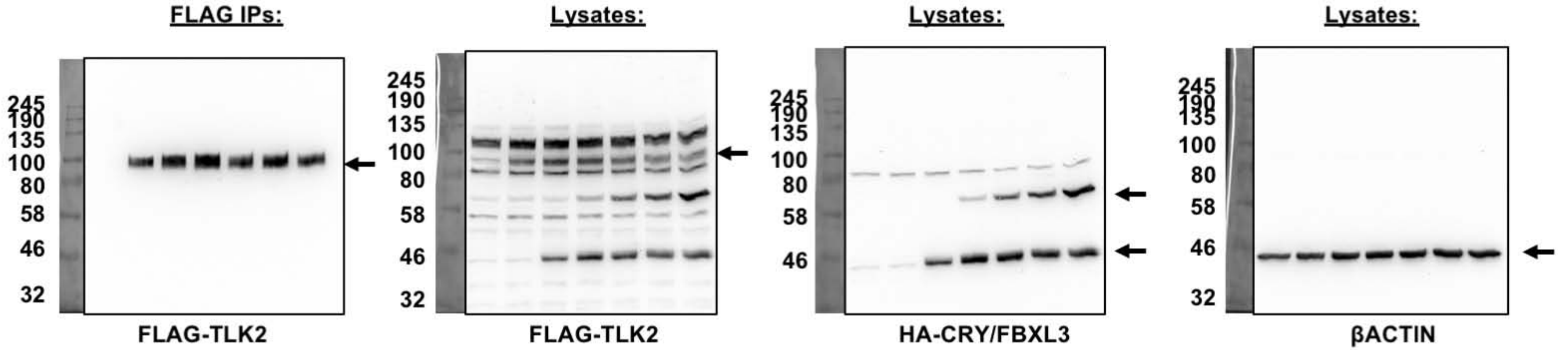

Supplementary Figure 13: Uncropped blots related to Figure 3C

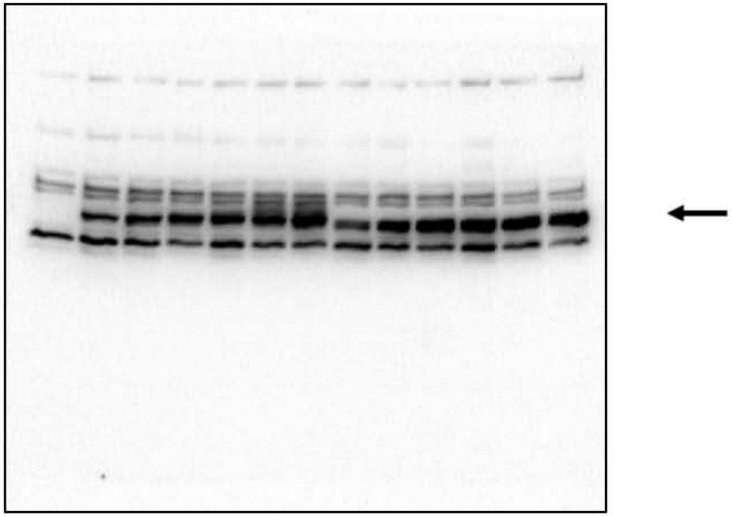

FLAG-TLK2

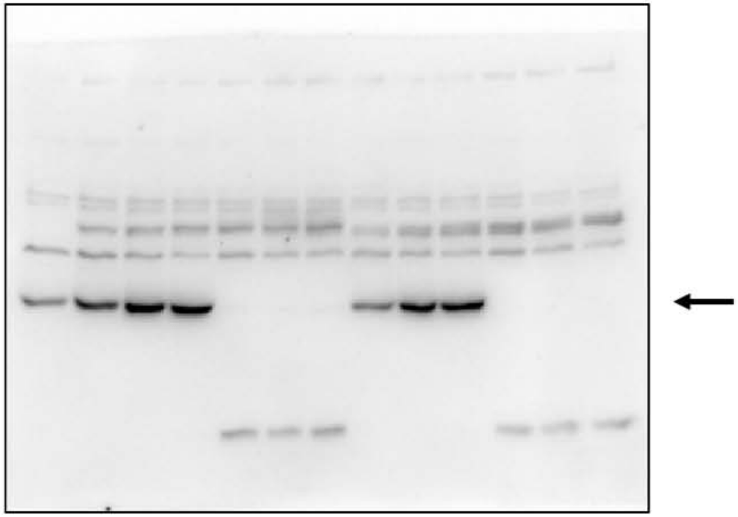

CRY1

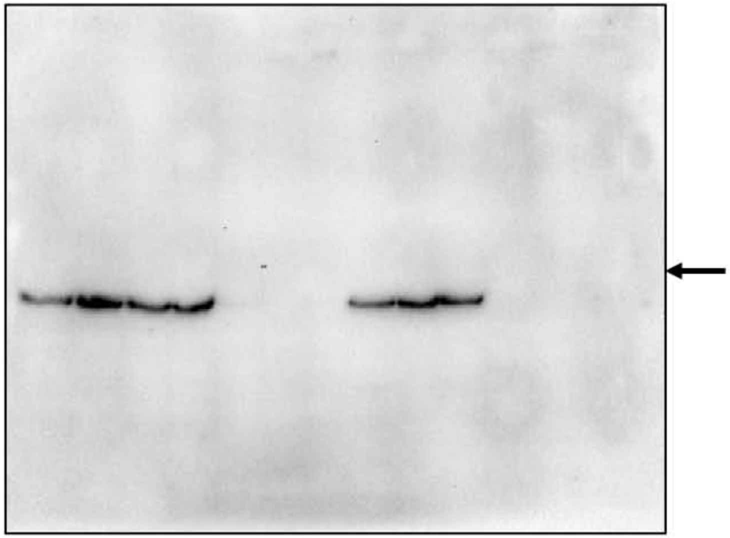

CRY2

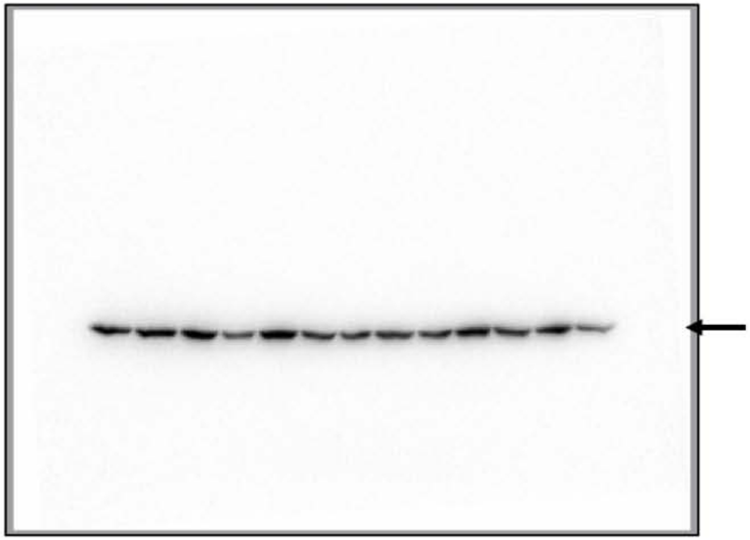

TUBULIN

Supplementary Figure 14: Uncropped blots related to Figure 4A

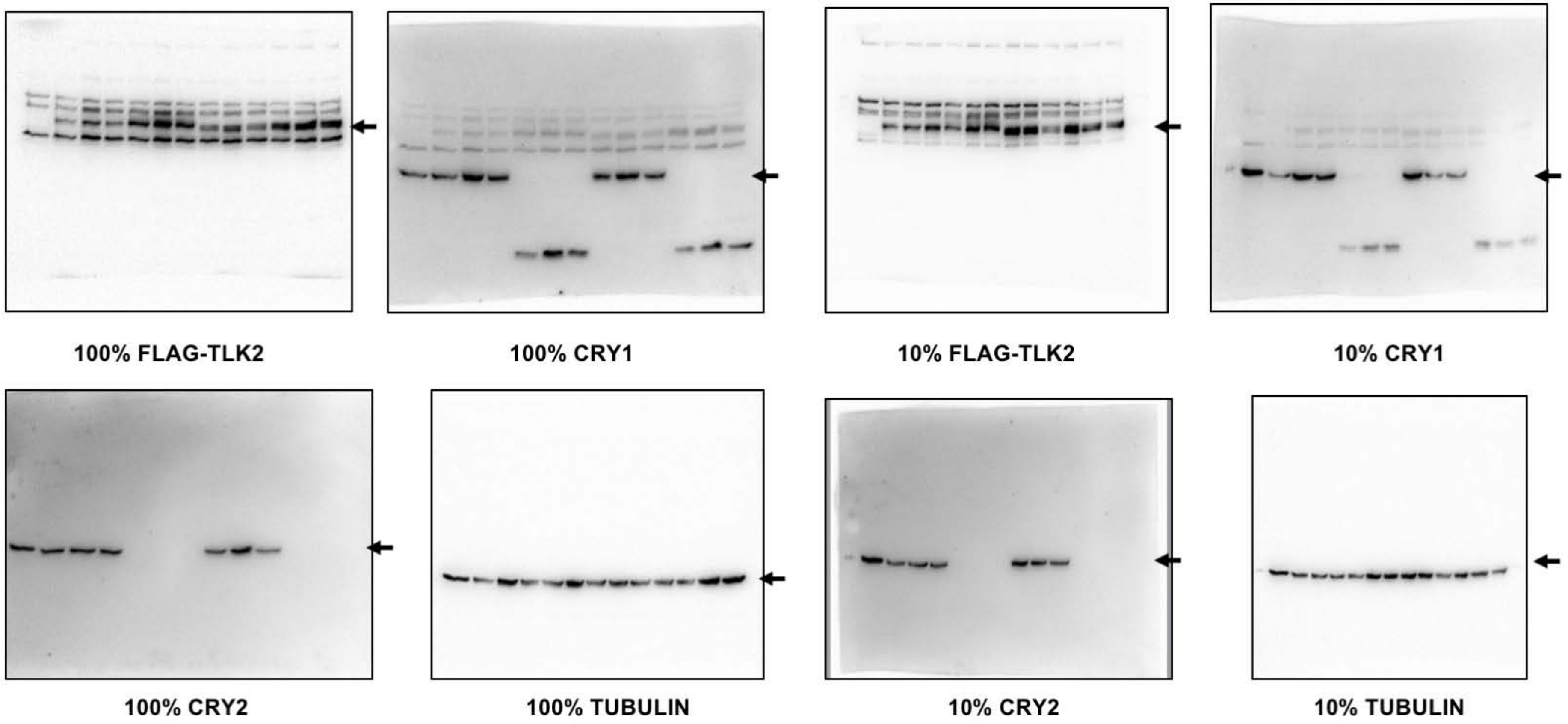

Supplementary Figure 15: Uncropped blots related to Figure 4B

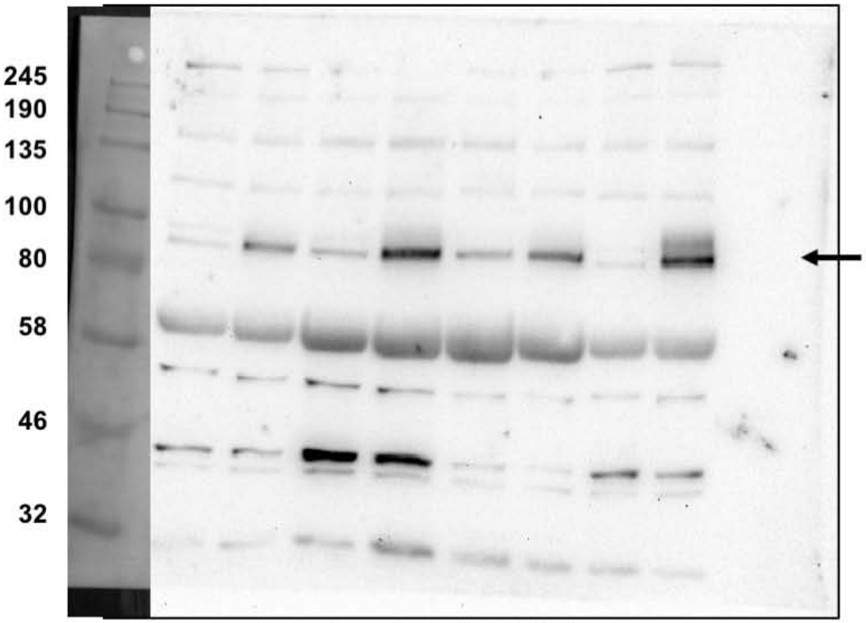

Experiment #1 FLAG-TLK2

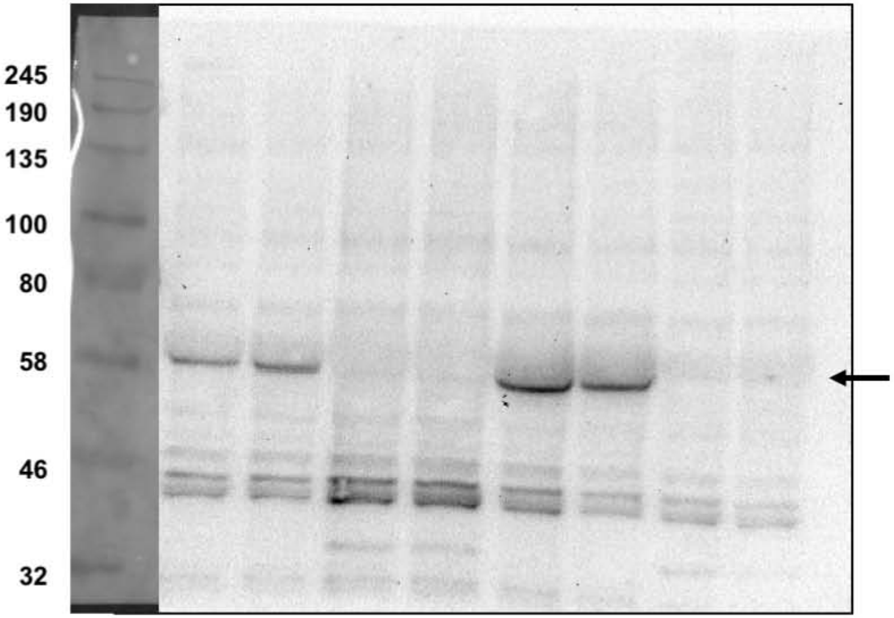

Experiment #1 CRY1

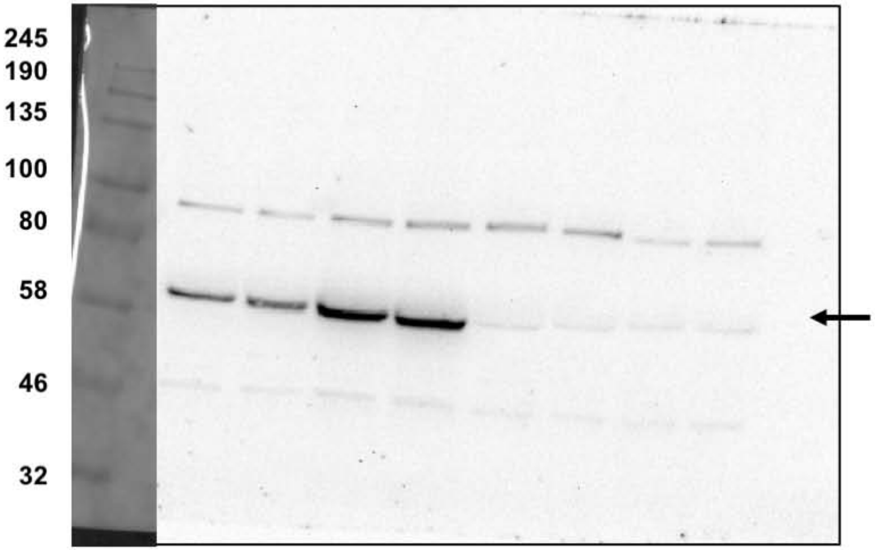

Experiment #1 CRY2

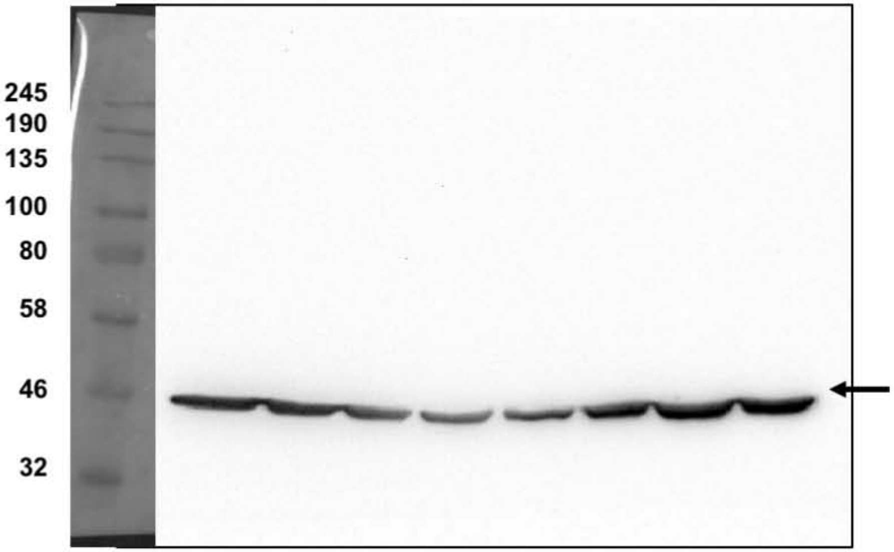

Experiment #1 ACTIN

Supplementary Figure 16: Uncropped blots related to Figure S5A

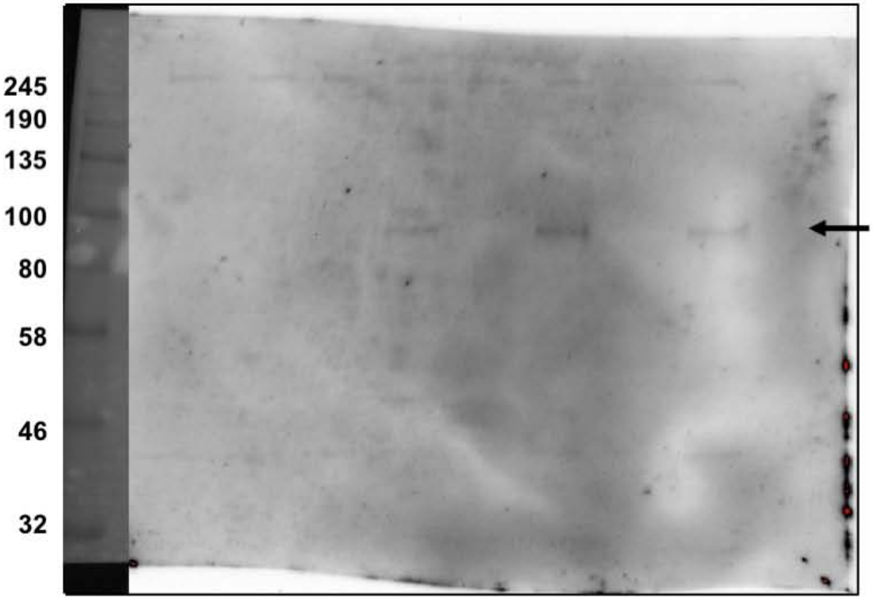

Experiment #2 FLAG-TLK2

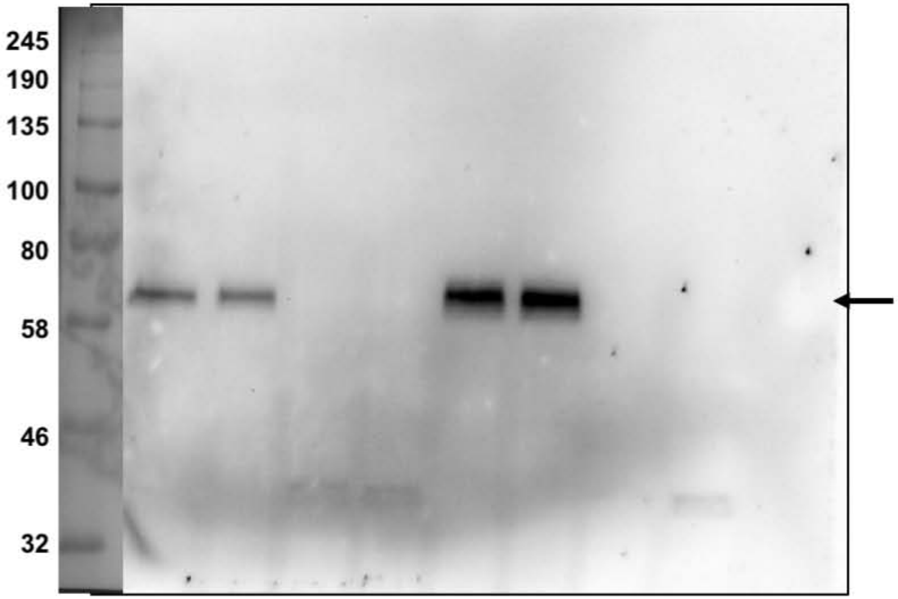

Experiment #2 CRY1

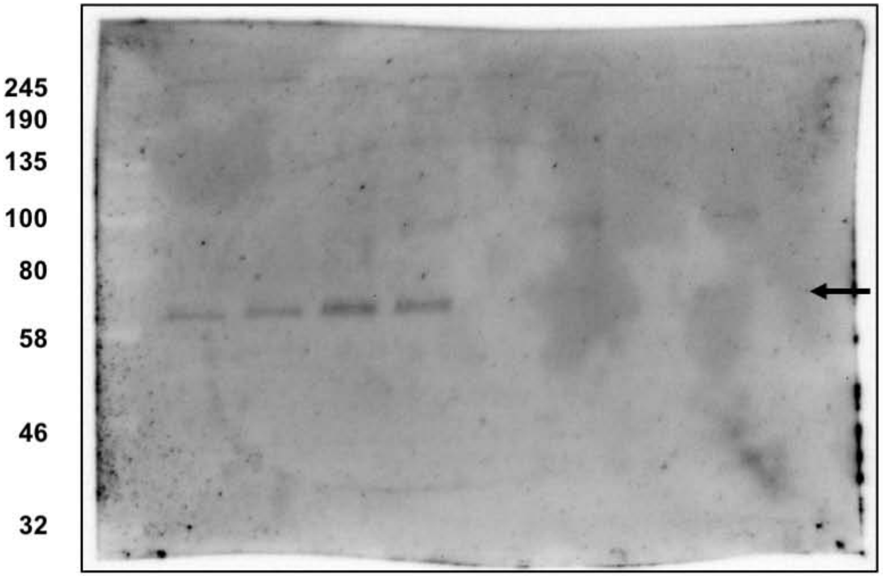

Experiment #2 CRY2

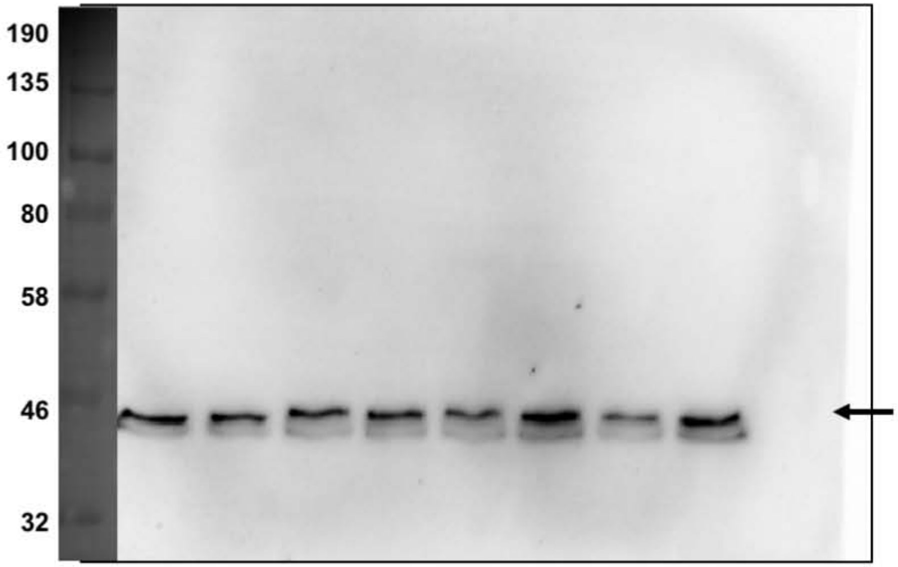

Experiment #2 ACTIN

Supplementary Figure 17: Uncropped blots related to Figure S5A

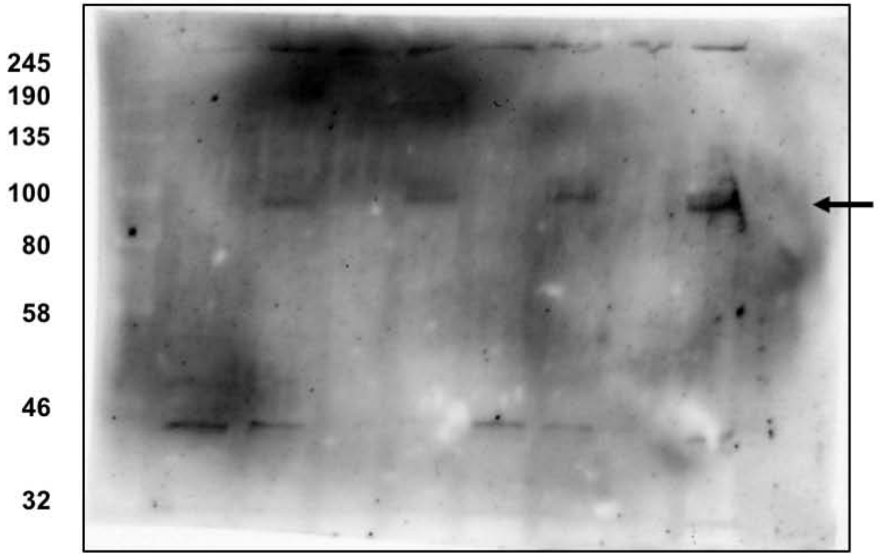

Experiment #3 FLAG-TLK2

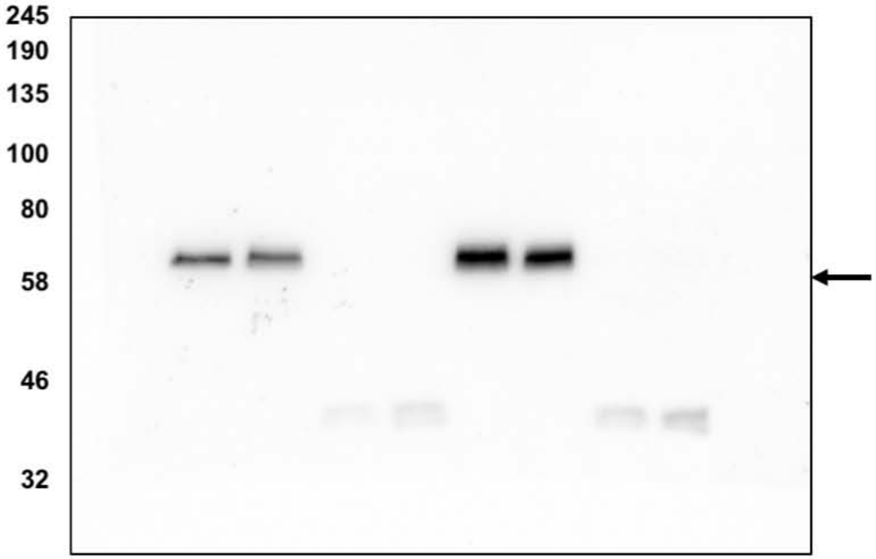

Experiment #3 CRY1

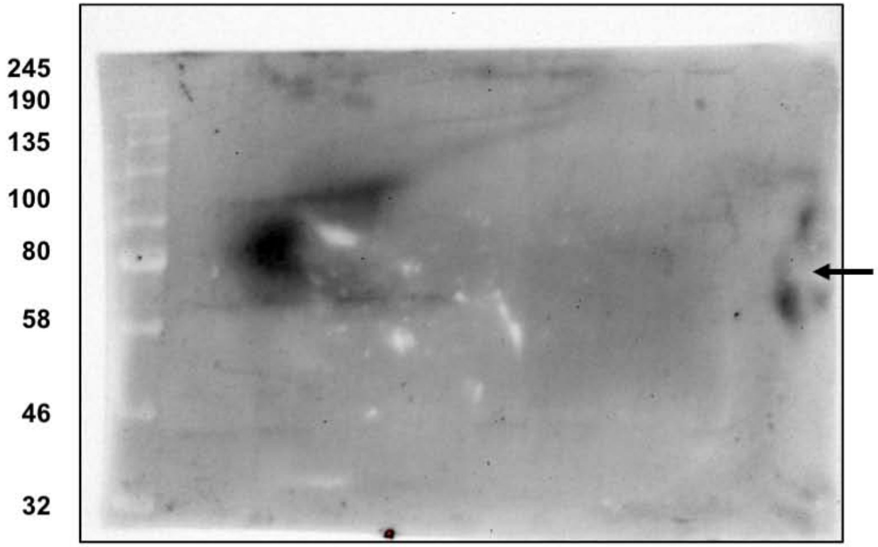

Experiment #3 CRY2

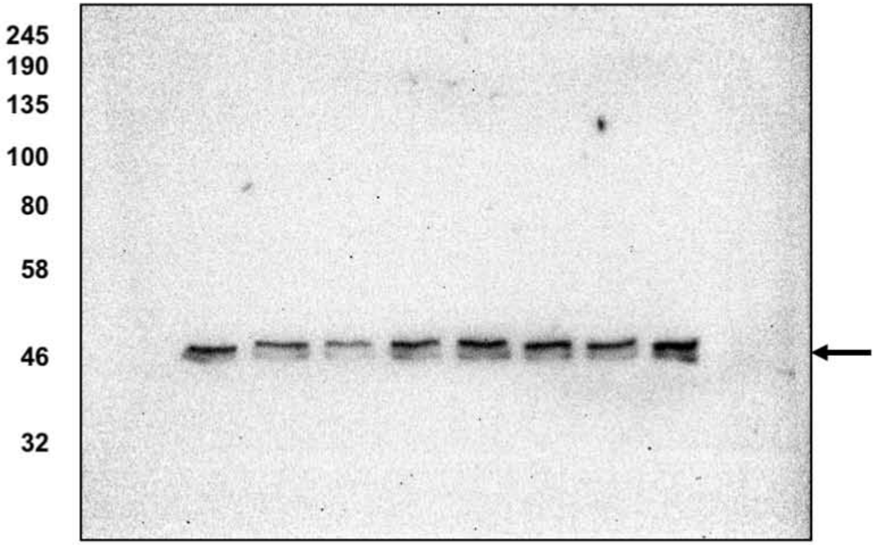

Experiment #3 ACTIN

Supplementary Figure 18: Uncropped blots related to Figure S5A

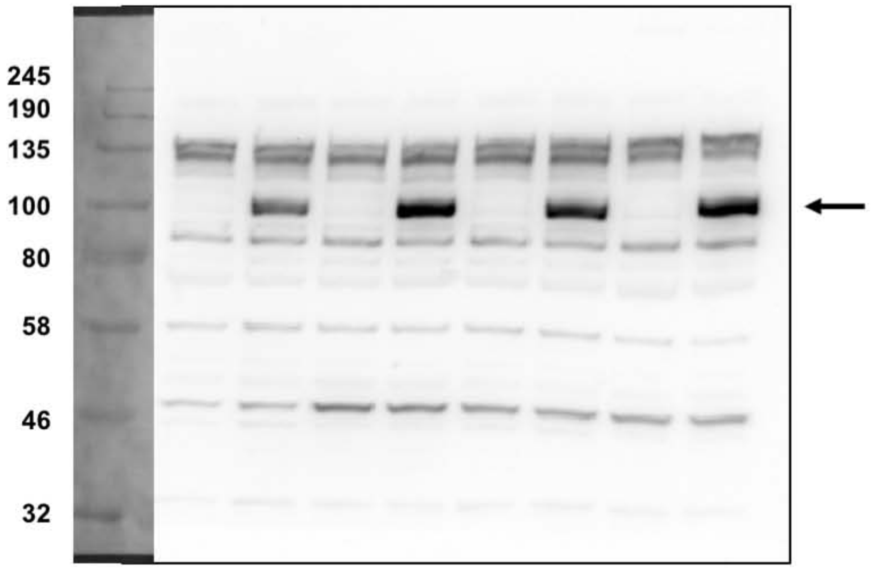

Experiment #1 FLAG-TLK2

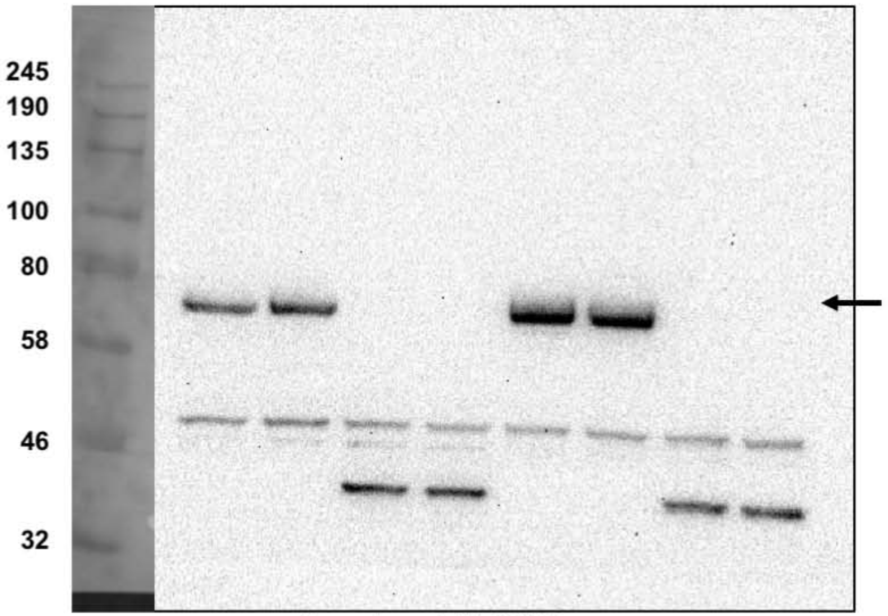

Experiment #1 CRY1

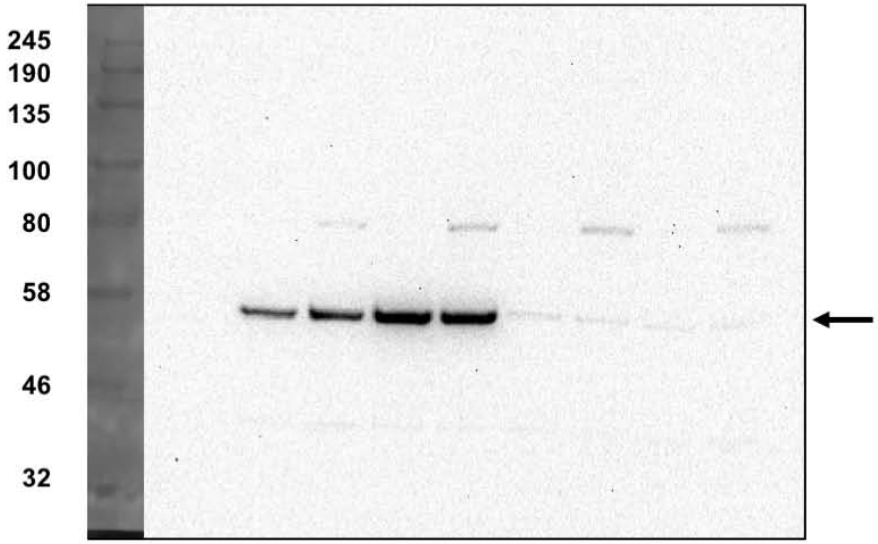

Experiment #1 CRY2

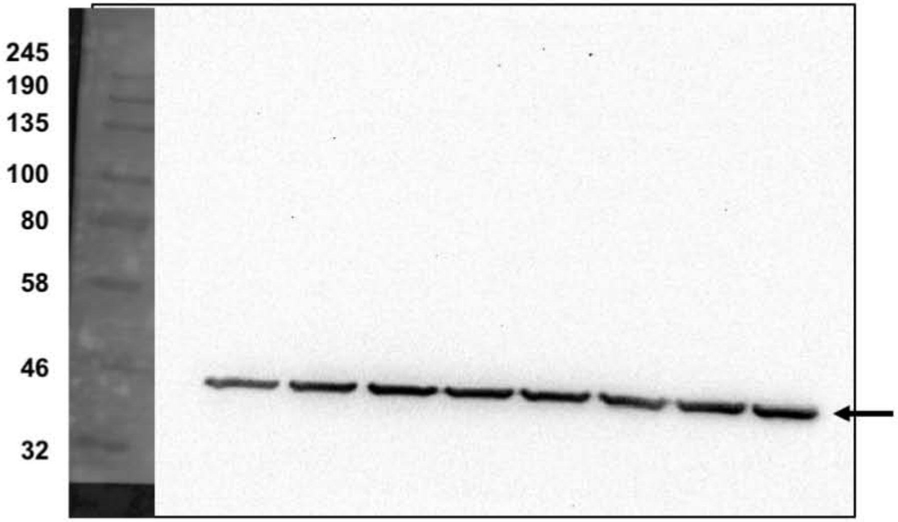

Experiment #1 ACTIN

Supplementary Figure 19: Uncropped blots related to Figure S5B

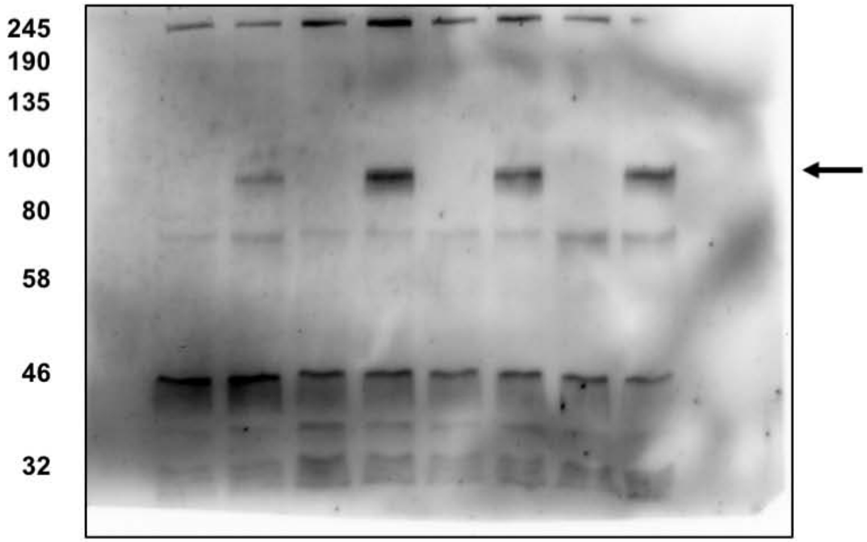

Experiment #2 FLAG-TLK2

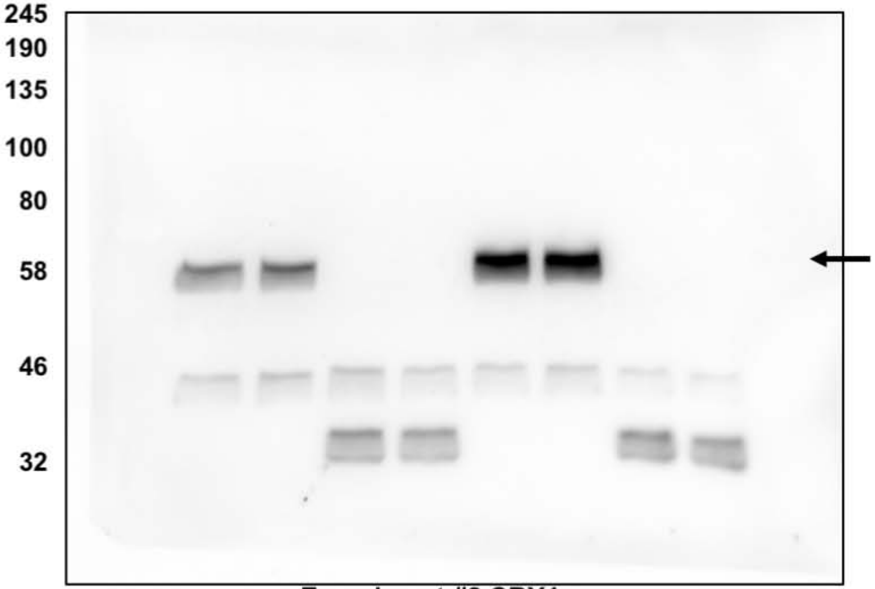

Experiment #2 CRY1

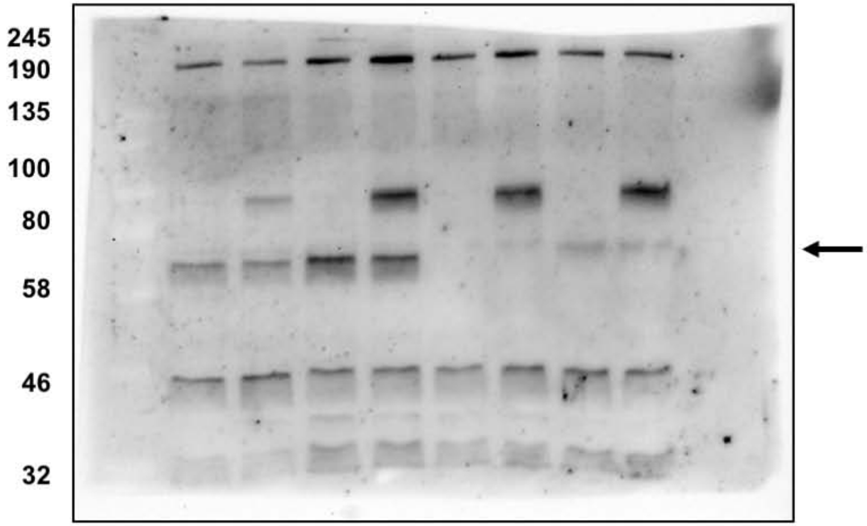

Experiment #2 CRY2

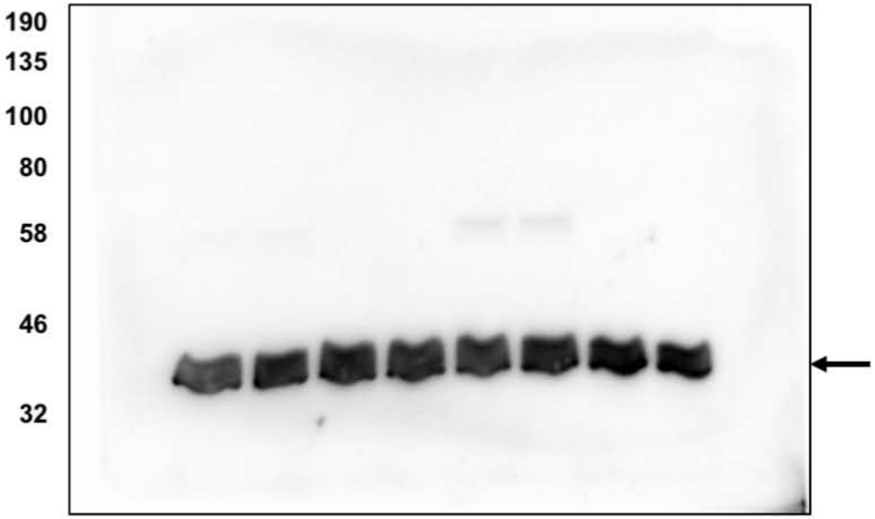

Experiment #2 ACTIN

Supplementary Figure 20: Uncropped blots related to Figure S5B

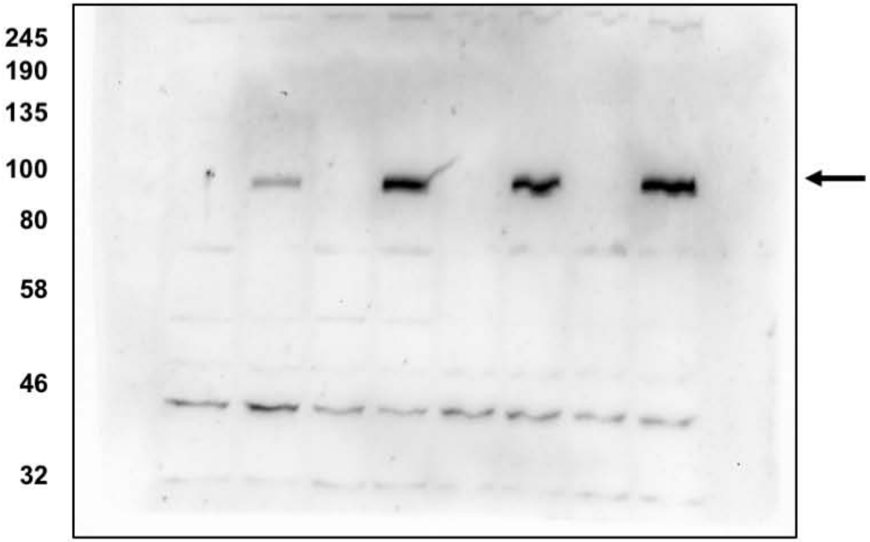

Experiment #3 FLAG-TLK2

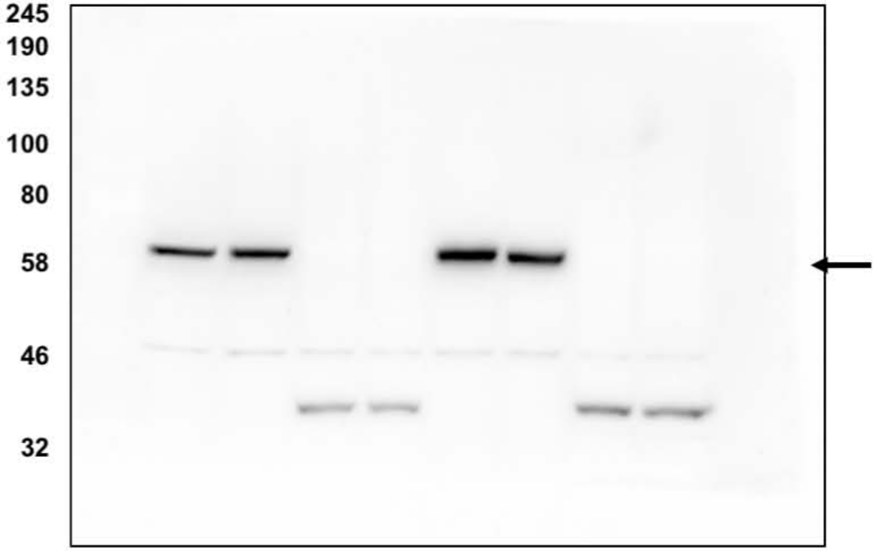

Experiment #3 CRY1

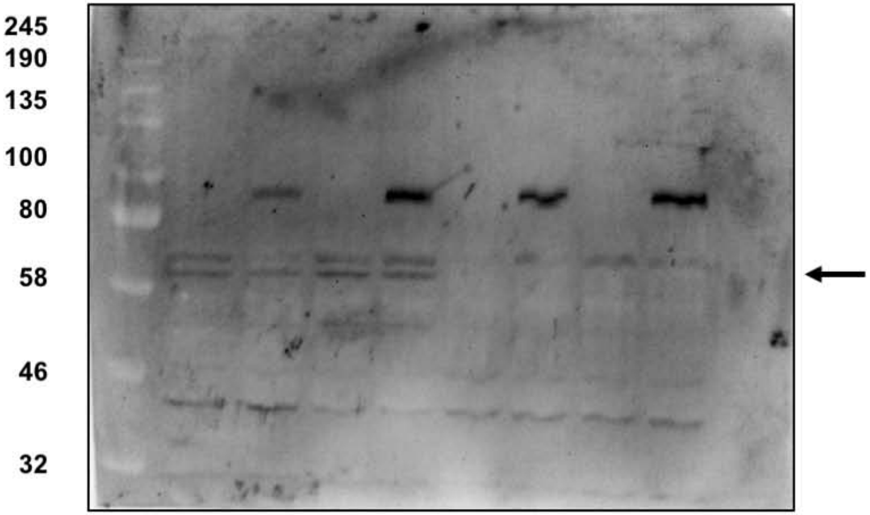

Experiment #3 CRY2

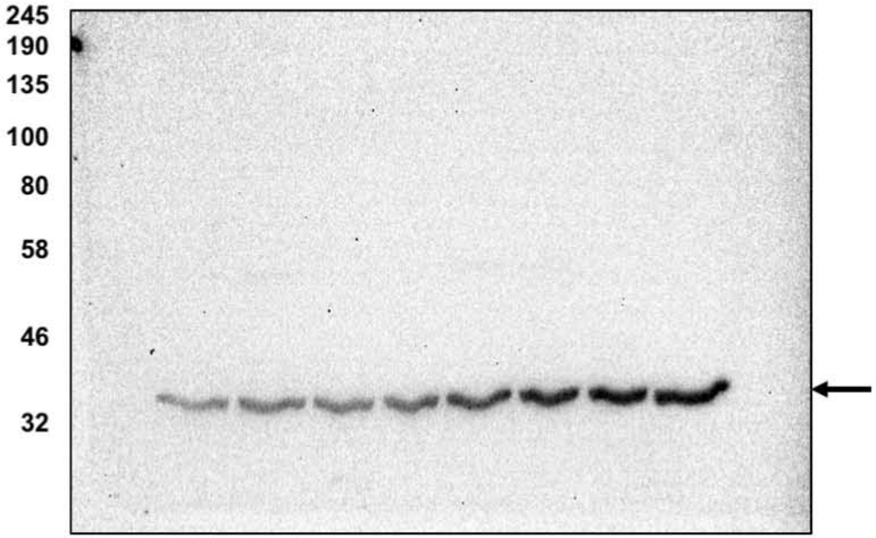

Experiment #3 ACTIN

Supplementary Figure 21: Uncropped blots related to Figure S5B
